# Design, synthesis and biological evaluation of novel orthosteric-allosteric ligands of the cannabinoid receptor type 2 (CB_2_R)

**DOI:** 10.3389/fchem.2022.984069

**Published:** 2022-09-27

**Authors:** Rebecca Ferrisi, Francesca Gado, Beatrice Polini, Caterina Ricardi, Kawthar A. Mohamed, Lesley A. Stevenson, Gabriella Ortore, Simona Rapposelli, Giuseppe Saccomanni, Roger G. Pertwee, Robert B. Laprairie, Clementina Manera, Grazia Chiellini

**Affiliations:** ^1^ Department of Pharmacy, University of Pisa, Pisa, Italy; ^2^ Department of Pharmaceutical Sciences, University of Milano Statale, Milan, Italy; ^3^ Department of Pathology, University of Pisa, Pisa, Italy; ^4^ College of Pharmacy and Nutrition, University of Saskatchewan, Saskatoon, SK, Canada; ^5^ School of Medicine, Medical Sciences and Nutrition, Institute of Medical Sciences, University of Aberdeen, Aberdeen, Scotland, United Kingdom; ^6^ Department of Pharmacology, College of Medicine, Dalhousie University, Halifax, NS, Canada; ^7^ CISUP, Centre for Instrumentation Sharing Pisa University, Pisa, Italy

**Keywords:** cannabinoid receptor type 2 (CB_2_R), CB_2_R allosteric modulators, dualsteric agents, antiinflammatory activity, human microglial cells

## Abstract

It is well known that G protein–coupled receptors (GPCRs) assume multiple active states. Orthosteric ligands and/or allosteric modulators can preferentially stabilize specific conformations, giving rise to pathway-biased signaling. One of the most promising strategies to expand the repertoire of signaling-selective GPCR activators consists of dualsteric agents, which are hybrid compounds consisting of orthosteric and allosteric pharmacophoric units. This approach proved to be very promising showing several advantages over monovalent targeting strategies, including an increased affinity or selectivity, a bias in signaling pathway activation, reduced off-target activity and therapeutic resistance. Our study focused on the cannabinoid receptor type 2 (CB_2_R), considered a clinically promising target for the control of brain damage in neurodegenerative disorders. Indeed, CB_2_R was found highly expressed in microglial cells, astrocytes, and even in some neuron subpopulations. Here, we describe the design, synthesis, and biological evaluation of two new classes of potential dualsteric (bitopic) CB_2_R ligands. The new compounds were obtained by connecting, through different linkers, the pharmacophoric portion of the CB_2_R positive allosteric modulator (PAM), **EC21a**, with that of the CB_2_R selective orthosteric agonist **LV62**, both developed in our laboratories. A preliminary screening enabled us to identify compound **JR64a** as the most promising of the series. Indeed, functional examination highlighted a signaling ‘bias’ in favor of G protein activation over βarrestin2 recruitment, combined with high affinity for CB_2_R and the ability to efficiently prevent inflammation in human microglial cells (HMC3) exposed to LPS/TNFα stimulation, thus demonstrating great promise for the treatment of neurodegenerative diseases.

## Introduction

Several neurodegenerative disorders display alterations in components of the endocannabinoid system (ECS), and a cannabinoid-based approach has proven efficacious in the reversal of certain neurodegenerative events in pre-clinical models of neuroinflammation, oxidative stress, and neuronal loss, among others (Basavarajappa et al., 2009; Di Marzo 2009; Aymerich et al., 2018; Mastinu et al., 2018). The ECS is a complex signaling system consisting of cannabinoid receptors, their endogenous ligands (known as “endocannabinoids”), and the enzymes responsible for endocannabinoid biosynthesis, cellular uptake and catabolism (Bisogno et al., 2005). The effects of endocannabinoids are primarily mediated by CB_1_ and CB_2_ cannabinoid receptors (CB_1_Rs and CB_2_Rs), which are G protein-coupled receptors (GPCRs), predominantly associated with Gα_i/o_ proteins (Howlett et al., 2002). Their activation inhibits adenylyl cyclase and certain voltage dependent calcium channels, and regulates mitogen-activated protein kinase (MAPK) and phosphatidylinositol-3-kinase (PI3K) pathways (Howlett et al., 2002). CB_1_Rs are highly expressed in the central nervous system (CNS) (Mackie 2005), where they play a well-established role in regulating neuronal excitability. In contrast, CB_2_Rs have reduced expression in the brain compared to CB_1_Rs. Indeed, the expression of CB_2_R in the brain has been mainly identified at the level of microglia and vascular elements (Walter et al., 2003; Ramirez et al., 2012), and their modulation is not accompanied by psychotropic side effects associated with the activation of CB_1_Rs.

Notably, activation of CB_2_Rs results in inhibition of neuroinflammatory signaling pathways (Bie et al., 2018); therefore, this receptor type may be a clinically promising target for the control of brain damage in neurodegenerative disorders, including neuropathic pain, Alzheimer’s disease, Parkinson’s disease, Huntington’s disease, and multiple sclerosis (Benito et al., 2003; Benito et al., 2005; Han et al., 2013; Chung et al., 2016; Xu et al., 2016; Ferrisi et al., 2021). However, to date only a few synthetic CB_2_R agonists have reached an advanced stage of clinical trials (from ClinicalTrials.gov: GW842166X, S-777469, and JTE-907), probably because of the predominance of CB_2_Rs on immune cells, whose activation might cause immunosuppression (Oláh et al., 2017).

Recent observations indicate that GPCRs are dynamic proteins able to assume multiple active states providing an interaction surface for intracellular adaptor proteins (e.g., heterotrimeric G proteins, G protein-coupled receptor kinases, and βarrestins) each of them responsible for different signaling pathway. Some ligands (orthosteric ligands or allosteric modulators) may stabilize a unique receptor conformation inducing a particular signaling pathway at the expense of others. This results in differential coupling to the signal transduction cascade and a biased response, a scenario which is also referred to as biased signaling or stimulus bias. Ligand bias should generate a biased response relatively independent of the cell system tested (Gurevich and Gurevich, 2019; Wootten et al., 2013; Smith et al., 2018). Biased GPCR ligands have been shown to display beneficial biological responses in preclinical and clinical studies, which explains the growing interests of medicinal chemists in biased signaling (Bock and Bermudez 2021). One of the most promising strategies to expand the repertoire of signaling-selective GPCR activators consists of dualsteric/bitopic agents, which are hybrid compounds composed of orthosteric and allosteric pharmacophoric units (Kamal and Jockers 2009; Lane et al., 2013). They bridge two topographically distinct ligand-binding domains, joining both orthosteric and allosteric properties within a single therapeutic agent. This strategy could offer access to GPCR modulators with a unique receptor subtype and signaling selectivity profile by virtue of targeting an allosteric site, as well as greater affinity due to the concomitant engagement with the orthosteric site. It is noteworthy that the special pharmacological profile of a bitopic ligand may be reflected in its unique binding kinetics. Theoretically, dualsteric ligands may have higher affinities than the respective partners. This may be derived from the ability of the two counterparts to bind into their corresponding binding pockets in an ideal manner without inducing an unfavorable conformational rearrangement of the receptor. In this view, upon binding, each pharmacophore has its own binding kinetics that can induce synergistic effects (i.e., allosteric cooperativity) on the overall kinetics of the bitopic ligand, greater than simply combining two individual components, as reported, for instance, for the M2 muscarinic receptor ligand, THR-160209 (Steinfeld et al., 2007). However, it may be expected that bitopic ligands do not always display an improved binding affinity; for example, a compromised binding affinity of the bitopic ligand can also occur when the individual pharmacophores have different preferences on receptor states (Antony et al., 2009). Bitopic/dualsteric ligands, may prove to be particularly useful in situations where endogenous agonist tone is progressively lost, such as in neurodegenerative disorders, thanks to the co-presence of the orthosteric and the allosteric modulator (Gentry et al., 2015). Finally, this innovative approach may also provide novel bias ligands because the incorporation of two pharmacophores in one ligand can severely impact receptor flexibility and thus signaling output (Lane et al., 2013; Schrage and Kostenis 2017; Reinecke et al., 2019).

**FIGURE 1:**
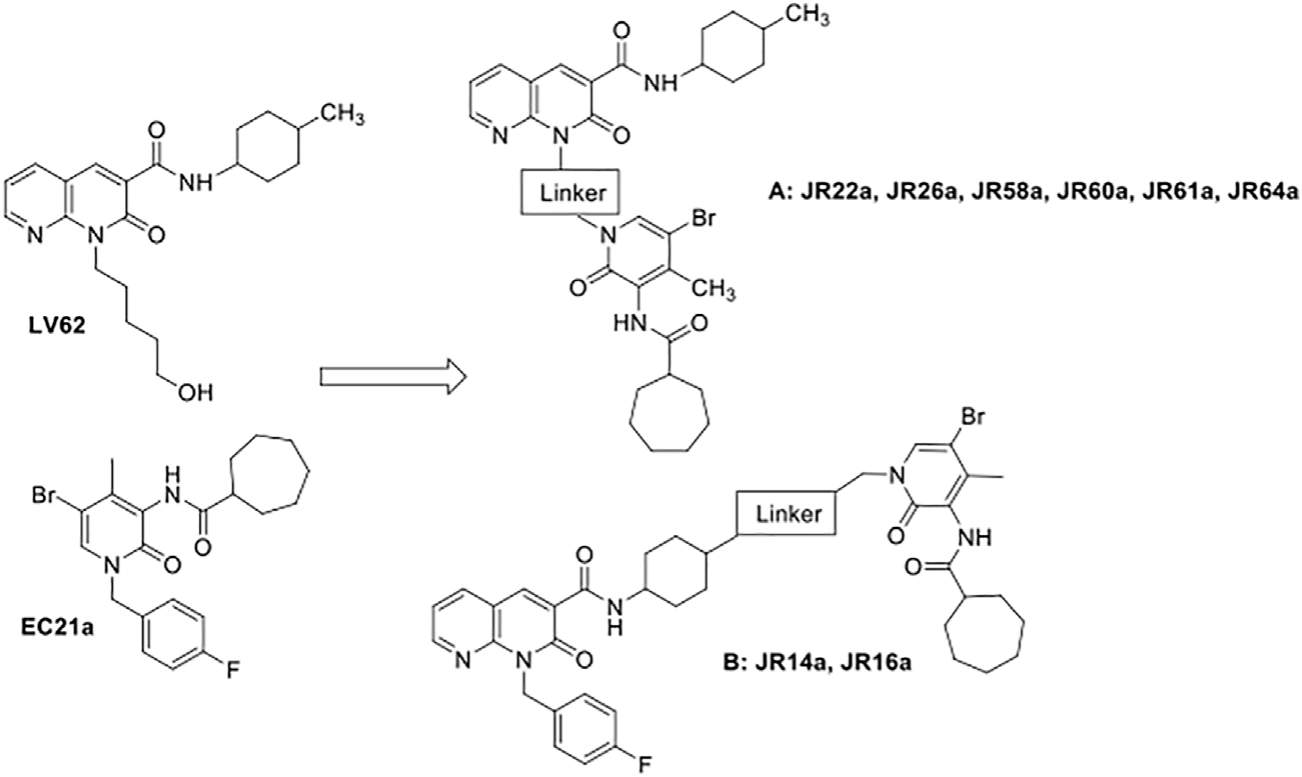
Design of potential CB_2_R dualsteric ligands **(A)** and **(B)**.

Here, we describe the design, synthesis, and biological evaluation of potential CB_2_R dualsteric ligands, characterized by general structures **A** and **B** (Figure 1). The two classes of compounds were obtained by connecting, through different linkers, the pharmacophoric portion of a previously identified CB_2_R positive allosteric modulator (PAM), namely **EC21a** (Gado et al., 2019; Shapiro et al., 2021), with that of the CB_2_R selective orthosteric agonist **LV62**, which belongs to the class of 1,8-naphthyridin-2(1*H*)-one-3-carboxamide derivatives, previously identified by us as potent CB_2_R orthosteric agonists (Lucchesi et al., 2014). The nitrogen atom in position 1 and the 4-methyl cyclohexyl group in position 3 of **LV62** moiety were selected to connect in position N (1) of the central core of **EC21a**, obtaining respectively the **A** (**JR22a, JR26a, JR58a, JR60a, JR61a, JR64a** compounds) and **B** (**JR14a, JR16a** compounds) series. Previous structural activity relation (SAR) studies on 1,8-naphthyridin-2(1*H*)-one-3-carboxamide derivatives (Lucchesi et al., 2014; Cooper et al., 2018) and on **EC21a** analogues (Gado et al., 2019; Gado et al., 2021) indicated these positions as the most suitable to chemical modifications without significantly compromising activity. The choice of the correct linker plays a crucial role in allowing the two pharmacophores to interact correctly with the respective binding site. Obviously, a deep knowledge of the orthosteric and allosteric binding sites will make easier the choice (Newman et al., 2020). The orthosteric CB_2_R binding site is well known now (Li et al., 2019), on the contrary there are no reliable structural data about the allosteric binding site of the CB_2_R. For this reason, the linkers used to obtain our ligands have been chosen on the basis of what previously reported for the bivalent/bitopic ligand (Kamal and Jockers. 2009; Newman et al., 2020; Meijer et al., 2021; Obeng et al., 2021; Gado et al., 2022).

A preliminary screening of the newly designed dualsteric CB_2_R ligands, led to the identification of compound **JR64a** as the most promising bitopic/dualsteric CB_2_R ligand of the series. Indeed, a signaling ‘bias’ in favor of G protein activation over βarrestin2 recruitment was observed, combined with high binding affinity for CB_2_R. Functional examination highlighted the ability of this compound to efficiently prevent inflammation in human microglial cells (HMC3) exposed to LPS/TNFα stimulation, as demonstrated by the reduced secretion of pro-inflammatory IL-6 and increased secretion of anti-inflammatory IL-10.

## Materials and methods

### Chemistry

Commercially available reagents were purchased from Sigma Aldrich, Tokyo Chemical Industry or Fluorochem and used without purification. ^1^H NMR and ^13^C NMR were recorded at 400 and 100 MHz respectively, on a Bruker AVANCE IIITM 400 spectrometer. Chemical shift (δ) is reported in parts per million related to the residual solvent signal, while coupling constants (J) are expressed in Hertz (Hz). All compounds are >95% pure by HPLC analysis. The analytical HPLC system consisted of a Varian 9,012 Solvent Delivery System, coupled to a Varian ProStar 330 DAD detector with operating wavelengths in the range between 200 and 400 nm, and the Star LC Workstation version 6.41 software was used for instrument control, data acquisition, and data processing. Analyses were performed on a reverse phase C18 column (Luna C18 (2) 150 mm × 4.6 mm, 5 µm particle size, Phenomenex^®^). The mobile phase was constituted by H_2_O (eluent A) and ACN (eluent B) at a flow rate of 600 μL/min. A linear gradient starting from 40% of A, changing to 80% of A over 30 min, and returning to the initial conditions over 20 min was used. The target compounds are ≥97% pure by HPLC analysis (Supporting Information). High resolution mass spectra (HRMS) were recorded on a Q Exactive™ Plus Hybrid Quadrupole-Orbitrap™ Mass Spectrometer (Thermo Fisher Scientific), equipped with HESI source. The ESI-MS spectrum was recorded by direct injection at 5 μml min- 1 flow rate. Working conditions: positive polarity, spray voltage 3.5 kV, capillary temperature 300°C, S-lens RF level 55, sheath gas 20, auxiliary gas 3 (arbitrary units); negative polarity, spray voltage 3.4 kV, capillary temperature 270°C, S-lens RF level 55, sheath gas 35, auxiliary gas 8 (arbitrary units). Acquisition and analysis: Xcalibur 4.2 software (Thermo). For spectra acquisition a nominal resolution (at m/z 200) of 140,000 was used. Organic solutions were dried over anhydrous Na_2_SO_4_. Evaporation was carried out in vacuo using a rotating evaporator. Silica gel flash chromatography was performed using silica gel 60 Å (0.040–0.063 mm; Merck Life Science S. r.l.). Reactions were monitored by TLC on Merck aluminium silica gel (60 F254) plates that were visualized under a UV lamp (*λ* = 254 nm). Melting points were determined on a Kofler hot-stage apparatus and are uncorrected.

The synthesis of the precursors of JR compounds is reported in Supporting Information.

**N-(4-(5-(4-(3-(5-bromo-3-(cycloheptanecarboxamido)-4- methyl -2-oxopyridin-1(2*H*)-yl)propyl)-1*H*-1,2,3-triazol-1-yl) pentanamido)cyclohexyl)-1-(4-fluorobenzyl)-2-oxo-1,2-dihydro-1,8-naphthyridine-3-carboxamide (JR14a).** To a solution of compounds **5** (40.0 mg, 0.08 mmol) and **15** (31.5 mg, 0.08 mmol) in DMF (1.71 ml) and water (0.85 ml), CuSO_4_.5H_2_O (20.0 mg, 0.08 mmol) and sodium ascorbate (47.6 mg, 0.24 mmol) were added. The reaction mixture was stirred at 80°C for 2 h. Subsequently, the solvent was removed under reduced pressure to give a residue which was dissolved in ethyl acetate and washed with saturated solution of NaHCO_3_. The organic phase was dried over Na_2_SO_4_, filtered, and evaporated under reduced pressure. The crude product obtained was purified by flash chromatography on silica gel using, firstly, ethyl acetate and 5% of methanol, and then ethyl acetate and 10% of methanol as eluent to afford compound **JR14a** (36.5 mg, 0.04 mmol) as a brownish solid. Yield: 50%. Mp: 108°C (dec.). ^1^H-NMR (CDCl_3_) δ (ppm) 9.90 (bd, 1H, *J =* 7.2 Hz, NH), 8.90 (s, 1H, H_4_), 8.72 (dd, 1H, *J =* 4.6 Hz; 1.8 Hz, H_7_), 8.10 (dd, 1H, *J =* 7.8 Hz; 1.8 Hz, H_5_), 7.55 (bs, 1H, NH), 7.43 (m, 3H, Ar-H + H_6_-Py), 7.37 (s, 1H, NCHC), 7.30 (dd, 1H, *J =* 7.8 Hz; 4.6 Hz, H_6_), 6.96 (AA’XX’ system, 2H, Ar-H), 5.78 (s, 2H, CH_2_), 5.63 (bd, 1H, *J =* 7.6 Hz, NH), 4.35 (t, 2H, *J* = 7.0 Hz, CH_2_-triazole), 4.20 (m, 1H, CHN), 3.99 (t, 2H, *J* = 7.0 Hz, CH_2_NCO), 3.93 (m, 1H, CH), 2.75 (t, 2H, *J* = 7.2 Hz, CH_2_-triazole), 2.51 (m, 1H, CHCO), 2.20 (t, 2H, *J =* 7.2 Hz, CH_2_CONH), 2.14 (s, 3H, CH_3_), 1.84 (m, 26H, cyclohexyl + cycloheptyl + 3xCH_2_). ^13^C-NMR (CDCl₃) δ (ppm) 176.11, 171.45, 162.87, 161.97, 162.22 (d, *J* = 245 Hz), 158.43, 152.30, 149.80, 146.50, 142.47, 142.23, 138.70, 133.10, 132.81 (d, *J* = 3 Hz), 130.29 (d, *J* = 8 Hz), 126.38, 123.31, 121.21, 119.56, 115.35 (d, *J* = 21 Hz), 115.10, 103.46, 49.92, 49.34, 47.85, 46.38, 45.54, 44.16, 35.78, 31.81, 29.72, 28.63, 28.55, 28.48, 28.24, 26.69, 22.56, 22.50, 20.43. HRMS-ESI: m/z calcd for C_46_H_55_BrFN_9_O_5_ [M-H]^-^, 910.34208; found 910.34450.

**N-(4-(5-(4-((5-bromo-3-(cycloheptanecarboxamido)-4-methyl-2-oxopyridin-1(2*H*)-yl)methyl)-1*H*-1,2,3-triazol-1-yl)pentanamido)cyclohexyl)-1-(4-fluorobenzyl)-2-oxo-1,2-dihydro-1,8-naphthyridine-3-carboxamide (JR16a).** Compound **JR16a** was prepared from compounds **5** and **16** as described for compound **JR14a** and purified by flash column chromatography on a silica gel using ethyl acetate and 10% of methanol. Yield: 55%. Mp: 113 °C (dec.). ^1^H-NMR (CDCl_3_) δ (ppm) 9.90 (bd, 1H, *J =* 7.6 Hz, NH), 8.91 (s, 1H, H_4_), 8.72 (dd, 1H, *J =* 4.8 Hz; 2.0 Hz, H_7_), 8.10 (dd, 1H, *J =* 7.8 Hz; 2.0 Hz, H_5_), 7.67 (s, 1H, NCHC), 7.64 (bs, 1H, NH), 7.46 (m, 3H, Ar-H + H_6_-Py), 7.31 (dd, 1H, *J =* 7.8 Hz; 4.8 Hz, H_6_), 6.97 (AA’XX’ system, 2H, Ar-H), 5.79 (s, 2H, CH_2_), 5.56 (bd, 1H, *J =* 7.6 Hz, NH), 5.15 (s, 2H, CCH_2_N), 4.35 (t, 2H, *J* = 7.4 Hz, CH_2_-triazole), 4.21 (m, 1H, CHN), 3.95 (m, 1H, CH), 2.50 (m, 1H, CHCO), 2.19 (t, 2H, *J =* 7.6 Hz, CH_2_CONH), 2.13 (s, 3H, CH_3_), 1.84 (m, 24H, cyclohexyl + cycloheptyl + 2xCH_2_). ^13^C-NMR (CDCl₃) δ (ppm) 176.14, 171.45, 162.85, 162.17 (d, *J* = 245 Hz), 161.96, 158.25, 152.32, 149.75, 143.33, 142.53, 142.03, 138.72, 133.06, 132.75 (d, *J* = 3 Hz), 130.19 (d, *J* = 9 Hz), 126.17, 123.90, 123.23, 119.58, 115.32 (d, *J* = 22 Hz), 115.08, 103.91, 50.15, 47.73, 46.28, 45.54, 44.52, 44.14, 35.67, 31.77, 29.55, 28.58, 28.52, 28.19, 26.66, 22.54, 20.37. HRMS-ESI: m/z calcd for C_44_H_51_BrFN_9_O_5_ [M-H]^-^, 882.31075; found 882,31,397.

**1-(5-(4-(3-(5-bromo-3-(cycloheptanecarboxamido)-4-methyl-2-oxopyridin-1(2*H*)-yl)propyl)-1*H*-1,2,3-triazol-1-yl)pentyl)-*N*-(4-methylcyclohexyl)-2-oxo-1,2-dihydro-1,8-naphthyridine-3-carboxamide (JR22a).** Compound **JR22a** was prepared from compounds **7** and **15** as described for compound **JR14a** and purified by flash column chromatography on a silica gel using ethyl acetate and 10% of methanol. Yield: 58%. Mp: 114 °C (dec.). ^1^H-NMR (CDCl_3_) δ (ppm) 9.96 and 9.60 (2d, 1H, *J =* 7.2 Hz, NH), 8.87 (s, 1H, H_4_), 8.70 (dd, 1H, *J* = 4.8 Hz; 2.0 Hz, H_7_), 8.08 (dd, 1H, *J* = 7.8 Hz; 2.0 Hz, H_5_), 7.47 (bs, 1H, NH), 7.44 (s, 1H, NCHC), 7.41 (s, 1H, H_6_-Py), 7.28 (dd, 1H, *J* = 7.8 Hz; 4.8 Hz, H_6_), 4.57 (2t, 2H, *J* = 7.6 Hz, CH_2_NCO), 4.38 (t, 2H, *J* = 7.2 Hz, CH_2_-triazole), 4.25 and 3.92 (2m, 1H, CH), 4.02 (t, 2H, *J* = 7.2 Hz, CH_2_NCO), 2.78 (t, 2H, *J* = 7.2 Hz, CH_2_-triazole), 2.50 (m, 1H, CHCO), 2.15 (s, 3H, CH_3_), 1.59 (m, 29H, cyclohexyl + cycloheptyl + 4xCH_2_), 0.97 and 0.92 (2d, 3H, *J* = 6.4 Hz, CH_3_). ^13^C-NMR (CDCl₃) δ (ppm) 176.10, 162.56, 161.96, 158.37, 152.12, 149.65, 146.46, 142.23, 142.00, 138.55, 133.09, 126.32, 123.14, 120.98, 119.15, 114.99, 103.44, 50.12, 49.36, 48.87, 47.77, 41.51, 33.94, 33.02, 32.04, 31.75, 29.96, 28.48, 28.18, 27.18, 26.64, 24.04, 22.46, 22.28, 20.42. HRMS-ESI: m/z calcd for C_40_H_53_BrN_8_O_4_ M-H]^-^, 787.33003; found 787.33307.

**1-(5-(4-((5-bromo-3- (cycloheptanecarboxamido)-4-methyl-2-oxopyridin-1(2*H*)-yl)methyl)-1*H*-1,2,3-triazol-1-yl)pentyl)-*N*-(4-methylcyclohexyl) -2-oxo-1,2-dihydro-1,8-naphthyridine-3-carboxamide (JR26a).** Compound **JR26a** was prepared from compounds **7** and **16** as described for compound **JR14a** and purified by flash column chromatography on a silica gel using ethyl acetate and 10% of methanol. Yield: 59%. Mp: 116 °C (dec.). ^1^H-NMR (CDCl_3_) δ (ppm) 9.98 and 9.59 (2d, 1H, *J =* 7.2 Hz, NH), 8.86 (s, 1H, H_4_), 8.69 (dd, 1H, *J* = 4.8 Hz; 2.0 Hz, H_7_), 8.07 (dd, 1H, *J* = 7.8 Hz; 2.0 Hz, H_5_), 7.66 (s, 1H, NCHC), 7.64 (bs, 1H, NH), 7.39 (s, 1H, H_6_-Py), 7.27 (dd, 1H, *J* = 7.8 Hz; 4.8 Hz, H_6_), 5.17 (s, 2H, CCH_2_N), 4.57 (2t, 2H, *J* = 7.6 Hz, CH_2_NCO), 4.35 (t, 2H, *J* = 7.4 Hz, CH_2_-triazole), 4.25 and 3.90 (2m, 1H, CH), 2.49 (m, 1H, CHCO), 2.14 (s, 3H, CH_3_), 1.56 (m, 27H, cyclohexyl + cycloheptyl + 3xCH_2_), 0.97 and 0.90 (2d, 3H, *J* = 6.6 Hz, CH_3_). ^13^C-NMR (CDCl₃) δ (ppm) 176.00, 162.62, 161.95, 158.16, 152.11, 149.73, 142.84, 142.00, 141.83, 138.51, 132.86, 126.13, 123.64, 123.28, 119.12, 114.99, 103.88, 50.41, 47.84, 45.74, 44.44, 41.42, 31.78, 31.09, 30.27, 29.87, 29.65, 28.17, 27.14, 26.66, 24.03, 21.58, 20.48. HRMS-ESI: m/z calcd for C_38_H_49_BrN_8_O_4_ M-H]-, 759.29873; found 759.29901.

**1-(5-(5-bromo-3-(cycloheptanecarboxamido)-4- methyl-2-oxopyridin-1(2H)-yl)pentyl)-N- (4-methylcyclohexyl)-2-oxo-1,2-dihydro-1,8-naphthyridine-3-carboxamide (JR61a).** NaH (60% dispersion in mineral oil) (8.4 mg, 0.21 mmol) was added portion wise at 0°C to a solution of compound **14** (70 mg, 0.21 mmol) in DME (0.4 ml) and anhydrous DMF (0.1 ml). LiBr (36.5 mg, 0.42 mmol) was added 15 min later. The mixture was stirred for 20 min at room temperature. Compound **6** (182 mg, 0.42 mmol), as a mixture of 1:1 *trans* and *cis* isomers, was added dropwise, and the reaction was stirred at 65°C overnight. Solvents were removed under reduced pressure. The mixture obtained was dissolved in CHCl_3_ and washed three times with water. The organic phase was dried over Na_2_SO_4_, filtered, and evaporated under reduced pressure. The crude product obtained was purified by flash chromatography on silica gel using hexane/AcOEt 5:5 as eluent to afford compound **JR61a** (33.0 mg, 0.05 mmol) as a yellowish solid. Yield: 24%. Mp: 118 °C (dec.). ^1^H-NMR (CDCl_3_) δ (ppm) 9.98 and 9.62 (2d, 1H, *J =* 7.8 Hz, NH), 8.85 (s, 1H, H_4_), 8.69 (dd, 1H, *J* = 4.4 Hz; 1.6 Hz, H_7_), 8.06 (dd, 1H, *J* = 7.8 Hz; 1.6 Hz, H_5_), 7.56 (bs, 1H, NH), 7.34 (s, 1H, H_6_-Py), 7.26 (dd, 1H, *J* = 7.8 Hz; 4.4 Hz, H_6_), 4.57 (2t, 2H, *J* = 7.6 Hz, CH_2_NCO), 4.24 and 3.89 (2m, 1H, CH), 3.91 (t, 2H, *J* = 7.4 Hz, CH_2_NCO), 2.50 (m, 1H, CHCO), 2.15 (s, 3H, CH_3_), 1.58 (m, 27H, cyclohexyl + cycloheptyl + 3xCH_2_), 0.96 and 0.90 (2d, 3H, *J* = 6.4 Hz, CH_3_). ^13^C-NMR (CDCl₃) δ (ppm) 175.95, 162.44, 161.82, 158.08, 151.95, 149.58, 141.80, 141.63, 138.38, 132.54, 126.27, 123.09, 118.96, 114.83, 103.35, 49.97, 48.69, 47.63, 45.56, 41.36, 33.80, 32.87, 31.88, 31.59, 30.94, 30.10, 29.49, 28.68, 28.03, 27.14, 26.49, 23.90, 22.14, 21.41, 20.32. HRMS-ESI: m/z calcd for C_35_H_46_BrN_5_O_4_ M-H]^-^, 678.26604; found 678.26887.

**1-(7-(5-bromo-3-(cycloheptanecarboxamido)-4-methyl-2-oxopyridin-1(2H)-yl)heptyl)-N-(4-methylcyclohexyl)-2-oxo-1,2-dihydro-1,8-naphthyridine-3-carboxamide (JR64a).** Compound **JR64a** was prepared from compounds **14** and **8** as described for compound **JR61a** and purified by flash column chromatography on a silica gel using hexane/AcOEt 5:5. Yield: 18%. Mp: 121 °C (dec.). ^1^H-NMR (CDCl_3_) δ (ppm) 10.01 and 9.65 (2d, 1H, *J =* 7.6 Hz, NH), 8.85 (s, 1H, H_4_), 8.69 (dd, 1H, *J* = 4.6 Hz; 1.8 Hz, H_7_), 8.06 (dd, 1H, *J* = 7.8 Hz; 1.8 Hz, H_5_), 7.49 (bs, 1H, NH), 7.31 (s, 1H, H_6_-Py), 7.26 (dd, 1H, *J* = 7.8 Hz; 4.6 Hz, H_6_), 4.54 (2t, 2H, *J* = 7.8 Hz, CH_2_NCO), 4.24 and 3.92 (2m, 1H, CH), 3.88 (t, 2H, *J* = 7.4 Hz, CH_2_NCO), 2.50 (m, 1H, CHCO), 2.15 (s, 3H, CH_3_), 1.60 (m, 31H, cyclohexyl + cycloheptyl + 5xCH_2_), 0.97 and 0.91 (2d, 3H, *J* = 6.4 Hz, CH_3_). ^13^C-NMR (CDCl₃) δ (ppm) 176.13, 162.68, 162.12, 158.25, 152.12, 149.84, 141.93, 141.75, 138.56, 132.65, 126.45, 123.33, 119.08, 115.05, 103.63, 50.29, 48.92, 47.93, 45.83, 42.03, 41.93, 34.03, 33.09, 32.12, 31.82, 30.32, 29.67, 29.30, 29.00, 28.25, 27.88, 26.99, 26.94, 26.71, 26.62, 22.36, 20.56. HRMS-ESI: m/z calcd for C_37_H_50_BrN_5_O_4_ M-H]^-^, 706.29734; found 706.29881.

**1-(2-(2-(5-bromo-3-(cycloheptanecarboxamido)-4-methyl-2-oxopyridin-1(2H)-yl)ethoxy)ethyl)-N-(4-methylcyclohexyl)-2-oxo-1,2-dihydro-1,8-naphthyridine-3-carboxamide (JR58a).** Compound **JR58a** was prepared from compounds **14** and **9** as described for compound **JR61a** and purified by flash column chromatography on a silica gel using hexane/AcOEt 3:7. Yield: 59%. Mp: 110 °C (dec.). ^1^H-NMR (CDCl_3_) δ (ppm) 9.95 and 9.60 (2d, 1H, *J =* 7.6 Hz, NH), 8.84 (s, 1H, H_4_), 8.60 (dd, 1H, *J* = 4.6 Hz; 2.0 Hz, H_7_), 8.02 (dd, 1H, *J* = 7.6 Hz; 2.0 Hz, H_5_), 7.66 (bs, 1H, NH), 7.31 (s, 1H, H_6_-Py), 7.25 (dd, 1H, *J* = 7.6 Hz; 4.6 Hz, H_6_), 4.80 (m, 2H, CH_2_NCO), 4.20 and 3.85 (2m, 1H, CH), 4.00 (m, 2H, CH_2_NCO), 3.80 (m, 2H, OCH_2_), 3.73 (m, 2H, OCH_2_), 2.48 (m, 1H, CHCO), 2.14 (s, 3H, CH_3_), 1.56 (m, 21H, cyclohexyl + cycloheptyl), 0.92 and 0.87 (2d, 3H, *J* = 6.4 Hz, CH_3_). ^13^C-NMR (CDCl₃) δ (ppm) 175.99, 162.68, 161.75, 158.15, 151.80, 149.75, 142.11, 141.98, 138.36, 133.99, 125.84, 123.33, 119.16, 114.74, 102.73, 68.43, 67.99, 49.35, 48.74, 47.60, 45.83, 40.48, 40.42, 33.79, 32.88, 31.88, 31.61, 30.10, 29.52, 28.03, 26.49, 22.10, 21.41, 20.24. HRMS-ESI: m/z calcd for C_34_H_44_BrN_5_O_5_ M-H]^-^, 680,24,530; found 680.24717.

**1-(4-(4-(5-bromo-3-(cycloheptanecarboxamido)-4-methyl-2-oxopyridin-1(2H)-yl)butoxy) butyl)-N-(4-methylcyclohexyl)-2-oxo-1,2-dihydro-1,8-naphthyridine-3-carboxamide (JR60a).** Compound **JR60a** was prepared from compounds **14** and **10** as described for compound **JR61a** and purified by flash column chromatography on a silica gel using hexane/AcOEt 3:7. Yield: 12%. Mp: 118 °C (dec.). ^1^H-NMR (CDCl_3_) δ (ppm) 10.01 and 9.65 (2d, 1H, *J =* 7.2 Hz, NH), 8.85 (s, 1H, H_4_), 8.66 (dd, 1H, *J* = 4.6 Hz; 1.4 Hz, H_7_), 8.05 (dd, 1H, *J* = 7.6 Hz; 1.4 Hz, H_5_), 7.51 (bs, 1H, NH), 7.34 (s, 1H, H_6_-Py), 7.24 (dd, 1H, *J* = 7.6 Hz; 4.6 Hz, H_6_), 4.61 (2t, 2H, *J* = 7.6 Hz, CH_2_NCO), 4.25 and 3.88 (2m, 1H, CH), 3.93 (t, 2H, *J* = 6.8 Hz, CH_2_NCO), 3.46 (m, 4H, 2xOCH_2_), 2.50 (m, 1H, CHCO), 2.14 (s, 3H, CH_3_), 1.58 (m, 29H, cyclohexyl + cycloheptyl, 4xCH_2_), 0.97 and 0.91 (2d, 3H, *J* = 6.4 Hz, CH_3_). ^13^C-NMR (CDCl₃) δ (ppm) 175.98, 162.50, 161.92, 158.15, 151.95, 149.67, 141.80, 141.62, 138.34, 132.70, 126.17, 123.07, 118.93, 114.84, 103.28, 70.42, 70.25, 49.83, 48.72, 47.66, 45.58, 44.91, 41.65, 33.81, 32.87, 31.90, 31.60, 30.09, 29.60, 28.03, 27.16, 27.12, 26.52, 26.49, 24.73, 22.60, 21.41, 20.24. HRMS-ESI: m/z calcd for C_38_H_52_BrN_5_O_5_ M-H]^-^, 736,30,788; found 736.30861.

### Cell cultures and reagents

CP55,940 was purchased from Cayman Chemicals (Ann Arbor, MI) [^3^H]CP55,940 at a concentration of 174.6 Ci/mmol was purchased from PerkinElmer (Guelph, ON). LPS (*Escherichia coli* 0111:B4) and TNFα were purchased from Sigma-Aldrich (Milan, Italy), whereas SR144528 was from Tocris (Bristol, United Kingdom). Chinese hamster ovary (CHO)-K1 cells originally obtained from ATCC (Catalog No. CCL-61, Manassas, VA) stably expressing human cannabinoid CB_1_R (*h*CB_1_R) or CB_2_R (*h*CB_2_R) described previously were maintained at 37°C, 5% CO_2_ in F-12 DMEM containing 1 mM l-glutamine, 10% FBS, and 1% Pen/Strep as well as hygromycin B (300 μg/ml) and G418 (600 μg/ml) for CHO-K1 *h*CB_1_R cells or G418 (400 μg/ml) for CHO-K1 *h*CB_2_R cells (Garai et al., 2020; Zagzoog et al., 2020). In the case of membrane collection for radioligand binding, cells were removed from flasks by scraping, centrifuged, and then frozen as a pellet at -80°C until required. Before use in a radioligand binding assay, cells were defrosted, diluted in Tris buffer (50 mM Tris [pH 7.4]) and homogenized with a 1 ml hand-held homogenizer. HitHunter^®^ (cAMP) and PathHunter^®^ (βarrestin2) CHO-K1 cells stably-expressing *h*CB_2_R from DiscoveRx (Eurofins, Fremont, CA) were maintained at 37 °C, 5% CO_2_ in F-12 DMEM containing 10% FBS and 1% Pen/Strep with 800 μg/ml geneticin (HitHunter^®^) or 800 μg/ml G418 and 300 μg/ml hygromycin B (PathHunter^®^), as previously reported (Garai et al., 2020; Zagzoog et al., 2020). The human microglial clone 3 cell line (HMC3) (ATCC^®^ CRL-3304™) was cultured in high glucose DMEM supplemented with 10% FBS, streptomycin (100 g/ml) and penicillin (100 U/mL) (Sigma-Aldrich, Milan, Italy).

### HitHunter^®^ cAMP assay

Inhibition of FSK-stimulated cAMP accumulation was measured using the DiscoveRx HitHunter assay. Twenty thousand cells/well were plated in low-volume 96-well plates and incubated overnight in Opti-MEM containing 1% FBS at 37°C and 5% CO_2_. Opti-MEM media was then removed and replaced with cell assay buffer (DiscoveRx) and cells were co-treated at 37°C with 10 µM FSK and ligands for 90 min cAMP antibody solution and cAMP working detection solutions were added to cells (DiscoveRx), and cells were incubated for 60 min at room temperature. cAMP solution A (DiscoveRx) was added, and cells were incubated for an additional 180 min at room temperature before chemiluminescence was measured on a Cytation5 plate reader (top read, gain 200, integration time 10,000 ms).

### PathHunter^®^ βarrestin2 assay

βarrestin2 recruitment was quantified using the DiscoveRx PathHunter^®^ assay. Cells (20,000 cells/well in low-volume 96-well plates) were incubated overnight in Opti-MEM containing 1% FBS at 37 °C and 5% CO_2_. Cells were treated with ligands for 90 min at 37 °C. Detection solution was added to cells (DiscoveRx), and cells were incubated for 60 min at room temperature. Chemiluminescence was measured on a Cytation5 plate reader (top read, gain 200, integration time 10,000 ms).

### Radioligand displacement assay

CHO-K1 cells were thawed, diluted in Tris buffer (50 mM Tris [pH 7.4]) and homogenized in a 1 ml hand-held homogenizer. *h*CB_1_R and *h*CB_2_R CHO-K1 cell membranes were collected by cavitation in a pressure cell, and sedimented by ultracentrifugation. Pellets were resuspended in TME buffer (50 mM Tris, 5 mM MgCl_2_, 1 mM EDTA [pH 7.4]), and protein concentration was measured via the Bradford method according to the manufacturer’s directions (Bio-Rad Laboratories, Mississauga, ON). Competition binding experiments were conducted with 1 nM [^3^H]CP55,940 and Tris binding buffer (50 mM Tris, 0.1% BSA [pH 7.4], 2 ml). Radioligand binding began with the addition of CHO-K1 cell membranes (25 µg protein per sample). Assays were performed for 120 min at 37°C and stopped by the addition of ice-cold Tris binding buffer (pH 7.4), followed by vacuum filtration using a 24-well sampling manifold (Brandel Cell Harvester; Brandel Inc., Gaithersburg, MD, United States ). Brandel GF/B filter paper was soaked with wash buffer at 4 °C for at least 24 h. Each filter paper was washed 6 times with a 1.2 ml aliquot of Tris-binding buffer (pH 7.4), then air-dried and submerged in 5 ml of scintillation fluid (Ultima Gold XR, PerkinElmer). Liquid scintillation spectrometry was used to quantify radioactivity. For competition binding experiments, specific binding was equal to the difference in radioactivity with or without 1 µM unlabelled CP55,940.

### Measurement of interleukins (IL-6 and IL-10) release in HMC3 cells

The concentrations of pro-inflammatory (IL-6) and anti-inflammatory (IL-10) interleukins were determined by performing specific ELISA assays (MyBioSource, San Diego, CA, United States ) on collected culture media. Human microglial cells (HMC3) were exposed to pretreatment with test compounds for 1 h followed by stimulation with LPS (10 μg/ml)/TNFα (50 ng/ml) for 24 h. In competition experiments, the CB_2_R antagonist (SR144528, 1 μM) or the CB_2_R positive allosteric modulator (**EC21a** 1 or 10 μM) were administered 15 min before agonist administration.

### Western blot analysis of CB2R expression in HMC3 cells

The expression of CB_2_Rs was evaluated in human microglia cell lysates by Western blot experiments. Briefly, 50 μg of protein was diluted with Laemmli sample buffer 2×, boiled for 8 min at 96°C, separated on Criterion TGXTM gel (4–20%) and transferred into PVDF membranes. To avoid non-specific immunodetection, membranes were incubated for 45 min in T-TBS (20 mM Tris, 500 mM NaCl, 0.1% Tween-20, pH 8) containing 5% non-fat milk. Blots were then incubated overnight at 4°C with a rabbit anti- CB_2_R antibody (#ab3561, Abcam, Cambridge, United Kingdom). Next, the membranes were incubated with HRP-labeled secondary anti-rabbit (#MAB201P, Merck-Millipore, Darmstadt, Germany) for 2 h at room temperature. Detection of chemiluminescence signals and densitometric analysis of blots were performed using ImageQuant LAS 4000 (GE Healthcare, Milan, Italy) and ImageLab software (Bio-Rad, Hercules, CA, United States ), respectively.

### Statistical analysis

Data for [^3^H]CP55,940 binding are expressed as % of maximum [^3^H]CP55,940 bound (i.e. 100%). HitHunter^®^ cAMP, and PathHunter^®^ βarrestin2 data are shown as % of maximal CP55,940 response (i.e. 100%). Concentration–response curves (CRC) were fit using non-linear regression (3 parameters) and used to calculate EC_50_, E_max_, or IC_50_ (GraphPad, Prism, v. 9.0). Statistical analyses were conducted by one-way analysis of variance (ANOVA), as indicated in the figure legends, using GraphPad. Post-hoc analyses were performed using Tukey’s (one-way ANOVA) test. Homogeneity of variance was confirmed using Bartlett’s test. All results are reported as the mean ± the standard error of the mean (SEM) or 95% confidence interval (CI), as indicated. *p*_values_ < 0.05 were considered significant.

## Results and discussion

### Chemistry

**SCHEME 1:**
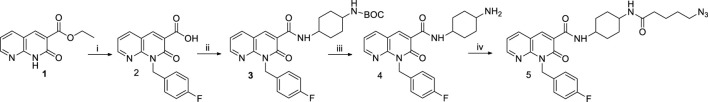
Synthetic pathway for the synthesis of the intermediate **5.** Reagents and conditions: i) a) DMF, Cs₂CO₃, 1h, rt; b) *p*-fluorobenzyl bromide, 50°C, 12h; c) NaOH 10%, 100°C, 24 h; ii) a) DMF, NEt₃, TBTU, 0°C, 30 min.; b) *cis*-4-(Boc-amino) cyclohexylamine, 0°C, 30 min.; c) rt, 12 h; iii) CH₂Cl₂, CF₃COOH, −20°C, 3h; iv) a) 5-azidopentanoic acid, DMF, NEt₃, TBTU, 0°C, 30 min.; b) compound **4**, 0°C, 30 min.; c) rt, 12h.

**SCHEME 2:**
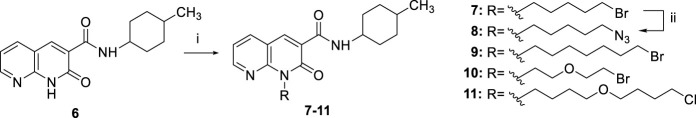
Synthetic pathway for the synthesis of the intermediates **7-11.** Reagents and conditions: *i*) a) CsF, DMF, rt, 1h; b) suitable R-Br or R-Cl, 30 °C, 24 h; *ii*) NaN_3_, DMF, 60 °C, 12 h.

**SCHEME 3:**
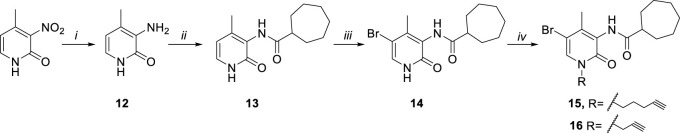
Synthetic pathway for the synthesis of the alkyne derivatives **15** and **16.** Reagents and conditions: i) Fe, NH_4_Cl, H_2_O/EtOH 1:2, 80 °C, 3 h. ii) a) cycloheptanecarboxylic acid, C_2_O_2_Cl_2_, DMF, rt, 0.5 h b) NEt_3_, DCM, DMF, rt, 24 h. iii) Br_2_, CHCl_3_, rt, 12 h. iv) a) CsF, DMF, rt, 1 h b) R-bromide, 30°C, 12h.

**SCHEME 4:**
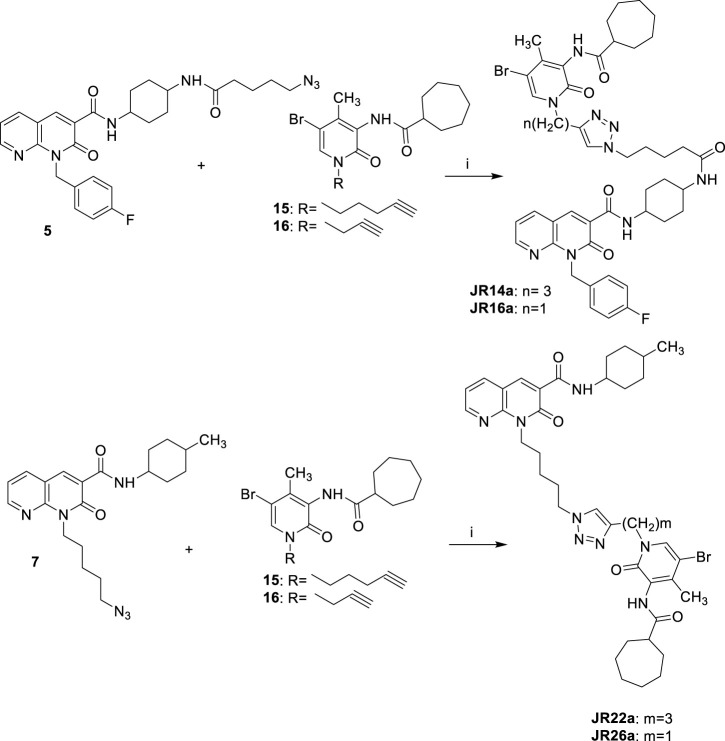
Synthetic pathway for the synthesis of compounds **JR14a, JR16a** and **JR22a**, **JR26a** Reagents and conditions: *i)* CuSO_4_·5H_2_O, sodium ascorbate, DMF/H_2_O 4:1, 80°C, 2 h.

**SCHEME 5:**
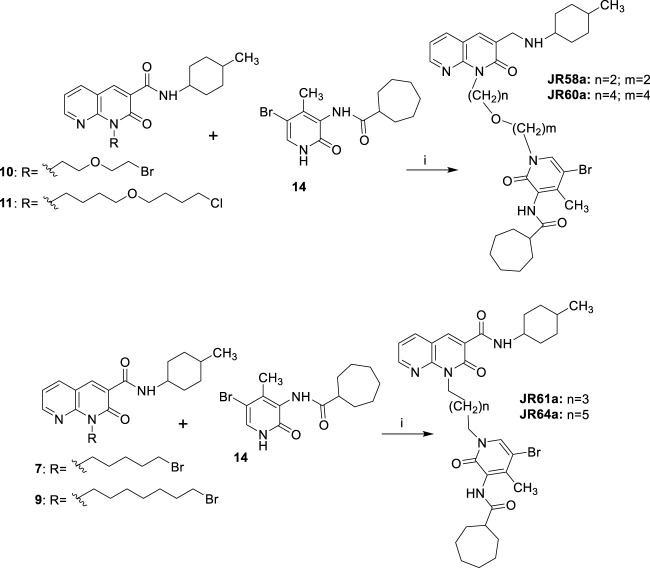
Synthetic pathway for the synthesis of compounds **JR58a**, **JR60a**, **JR61a** and **JR64a**. Reagents and conditions: *i)* a) NaH, DME, DMF, 0°C, 30 min; b) LiBr r.t. 20 min; suitable c) 65 °C, 12 h.

The synthesis of novel compounds, **JR14a**, **JR16a**, **JR22a**, **JR26a**, **JR58a**, **JR60a**, **JR61a** and **JR64a**, along with their corresponding precursors, was accomplished as depicted in Schemes 1-5.

As described in Scheme 1, the ethyl ester **1** (Manera et al., 2009) was dissolved in anhydrous DMF with the *p*-fluorobenzyl bromide in the presence of cesium carbonate at 50 °C for 12 h. After this time and solvent removal, the crude product was immediately subjected to alkaline hydrolysis of ester group in the presence of NaOH al 10% at reflux for 24h, followed by acidification, affording the carboxylic acid **2**. The amide **3** was obtained by reaction of derivative **2** with *cis*-4-(Boc-amino) cyclohexylamine in the presence of TBTU, NEt_3_, at room temperature for 12 h. The tert-butyl carbamate (BOC) deprotection of **3** was performed under acidic conditions by using CF₃COOH at −20°C for 3 h to yield the amino-derivative **4**. Finally, the derivative **4** was reacted with 5-azidopentanoic acid (previously prepared by reacting 5-bromovaleric acid, with NaN₃ in DMF at 60°C for 12 h) in the presence of TBTU according to the procedure described above, to afford the desired compound **5** (Scheme 1).

As reported in Scheme 2, the N1-alkylation of 1,8-naphthyridine-3-carboxamide **6** [(Manera et al., 2009) in anhydrous DMF with the suitable halogenated reagent in the presence of cesium fluoride afforded the desired 1,8-naphthyridin-2-one derivatives **7, 9–11**. Additionally, compound **7** was also used as starting material in the presence of sodium azide at 60°C for 12 h to give compound **8**.

As reported in Scheme 3, the commercially available starting material 2-hydroxy-4-methyl-3-nitropyridine was treated with iron powder and ammonium chloride at 80°C for 3 h, to afford the amine compound **12**. The reaction between the amine derivative **12** and the cycloheptanecarbonyl chloride in DMF and triethylamine initially at 0°C and then at room temperature for 24 h, gave the amides **13**. The acyl chloride was prepared by reaction between cycloheptanecarboxylic acid and oxalyl chloride at room temperature for 30 min. The 5-bromo derivative **14** was obtained from compound **13** for treatment with Br_2_ in CHCl_3_ at room temperature for 12 h. Finally, compound **14** was subjected to a N-alkylation reaction by treatment with cesium fluoride in anhydrous DMF, at room temperature for 1 h and then with the suitable halogenated reagent at 50°C for 12 h, affording the desired alkyne derivatives **15** and **16**.

As showed in Scheme 4, the final compounds **JR14a** and **JR16a** were easily obtained by click chemistry reaction of the azido derivative **5** with the alkyne derivatives **15** or **16** in DMF and water in the presence of CuSO_4_.5H_2_O and sodium ascorbate, at 80°C for 2 h. The same click reaction was also conducted between the azido derivative **7** with the alkyne derivatives **15** and **16** to afford the compounds **JR22a** and **JR26a.**

Finally, the synthesis of final compounds **JR58a**, **JR60a**, **JR61a** and **JR64a,** was accomplished as depicted in Scheme 5. The 5-bromo derivative **14** was subjected to a N-alkylation reaction by treatment with sodium hydride at 60% in anhydrous DME: DMF (4:1, v/v) mixture, at 0°C for 15 min, followed by the addition of lithium bromide, at room temperature for 20 min, and then of the appropriate N1-alkylated intermediate **7, 9–11** at 65 °C for 12 h, affording, after purification, the desired compounds **JR58a**, **JR60a**, **JR61a** and **JR64a**.

### Gα_i/o_ protein-dependent inhibition of forskolin (FSK)-stimulated cAMP accumulation and ligand induced recruitment of βarrestin2

**FIGURE 2:**
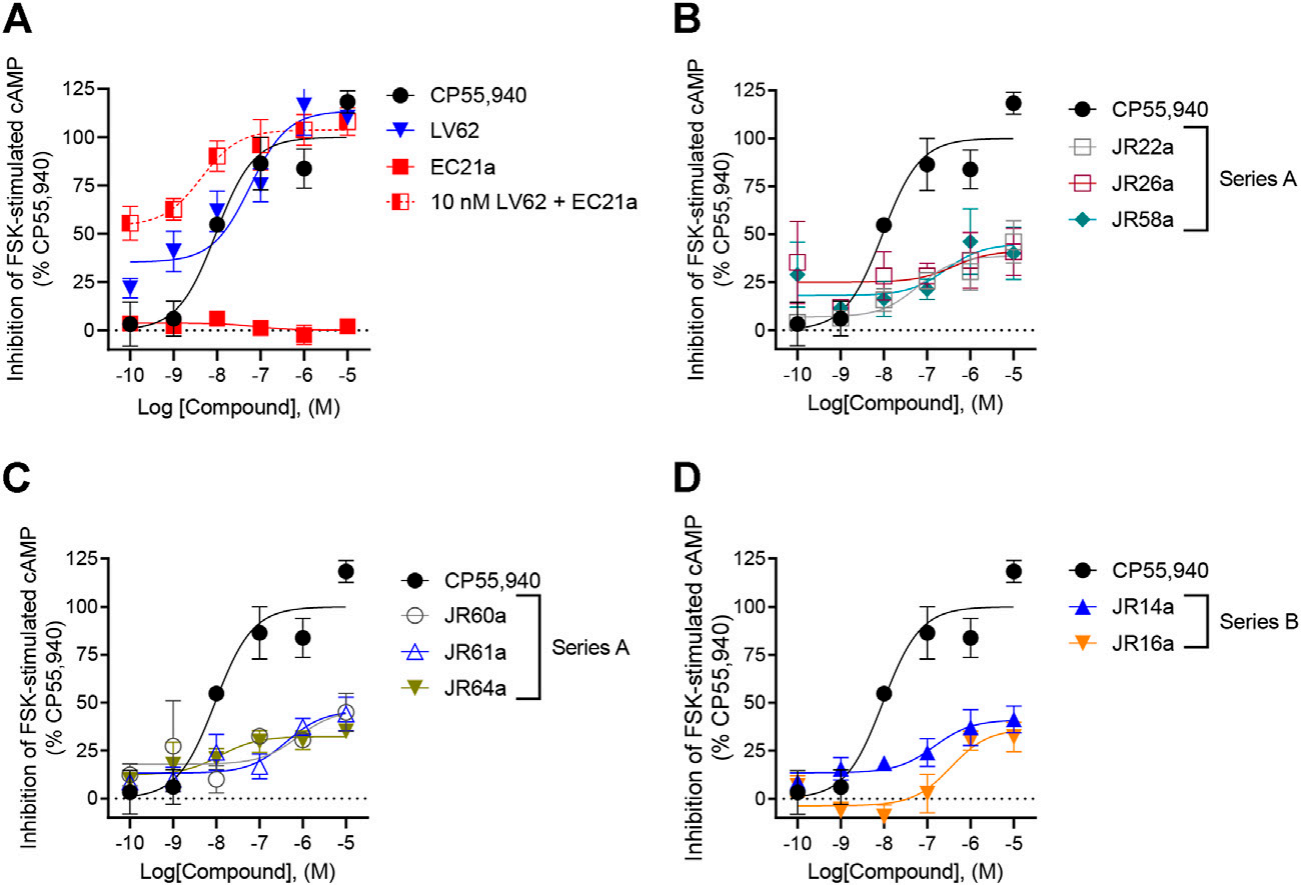
Evaluation of *h*CB_2_R-dependent inhibition of FSK-stimulated cAMP. CHO cells stably-expressing *h*CB_2_R were treated with 0.10 nM—10 μM compounds and 10 µM FSK for 90 min and cAMP inhibition was measured. **(A)** Parent compounds used for the design of the JR derivatives; **(B,C)** compounds of series **A**; **(D)** compounds of series **B**. CP55,940 data are the same in all four panels and presented for reference. Data are expressed as % CP55,940 response. Data were fitted to a non-linear regression (3 parameter model, GraphPad v. 9.0). Data are mean ± S.E.M. of six independent experiments performed in triplicate.

The new dualsteric CB_2_R ligands of general chemical structure **A** (**JR22a**, **JR26a**, **JR58a**, **JR60a**, **JR61a**, and **JR64a**) and **B** (**JR14a**, **JR16a)**, along with their relative parent compounds **EC21a** and **LV62,** were screened to assay their ability to inhibit FSK-stimulated cAMP accumulation in CHO-K1 cells stably-expressing *h*CB_2_R (Table 1). The non-selective orthosteric CBR ligand CP55,490 was used as a reference compound. Cells were treated with 10 μM FSK and CP55,490 or compound for 90 min to assess ligand concentration-dependent activity. Regarding the parent compounds, for **EC21a** no response was detected in accordance with its allosteric nature. **LV62** showed nanomolar potency (EC_50_ = 58 nM) and high relative efficacy (E_max_ = 110 ± 8.8) (Table 1; Figure 2A). The baseline for **LV62** was higher than that of CP55,940 (Figure 2A), indicating that *E*_min_ and *E*_max_ may be poorly defined within this assay, which may be a product of the curve fit for **LV62** or an artefact of the assay. In co-administration experiments, the activity of 10 nM **LV62** was augmented by **EC21a** in a concentration-dependent manner (EC_50_ = 4.3 nM) (Table 1; Figure 2A), confirming the role of **EC21a** as a CB_2_R positive allosteric modulator (PAM) (Gado et al., 2019).

**TABLE 1:** Inhibition of forskolin-stimulated cAMP and βarrestin2 recruitment.

Compound(s)	Inhibition of cAMP		βarrestin2 recruitment	
	EC_50_ (nM) (95% C.I.)	*E*_max_ (% CP55,940) ± S.E.M	EC_50_ (nM) (95% C.I.)	*E*_max_ (% CP55,940) ± S.E.M
CP55,940	9.4 (3.4–29)	100 ± 6.4	560 (410–760)	100 ± 3.4
EC-21a	>10,000	2.5 ± 0.53*	>10,000	1.4 ± 0.96*
LV62	58 (5.5–250)	110 ± 5.3	63 (49–82)*	65 ± 1.4*
10 nM LV62 + EC21a	4.3 (0.47–34)	104 ± 5.2	n.d	n.d
JR14a	150 (51–220)	41 ± 5.7*	25 (16–39)*	39 ± 0.93*
JR16a	350 (78–850)*	36 ± 8.6*	n.c	27 ± 4.2*
JR22a	62 (2.5–410)	38 ± 5.5*	>10,000	33 ± 3.1*
JR26a	110 (60–210)*	41 ± 5.1*	>10,000	39 ± 6.1*
JR58a	270 (36–440)*	45 ± 2.9*	>10,000	51 ± 2.8*
JR60a	770 (200–1,110)*	46 ± 4.4*	>10,000	36 ± 9.2*
JR61a	420 (14–630)	46 ± 1.7*	>10,000	34 ± 2.2*
JR64a	8.6 (5.9–12)	38 ± 0.74*	>10,000	45 ± 2.4*

*h*CB_2_R activity was quantified for cAMP inhibition using the DiscoveRx HitHunter^®^ assay (CHO-K1 *h*CB_2_R) in cells treated with compounds for 90 min, and for βarrestin2 recruitment using the DiscoveRx PathHunter^®^ assay (CHO-K1 *h*CB_2_R) in cells treated with compounds for 90 min. Data were fit to a variable slope (3 parameter) non-linear regression in GraphPad (v. 9.0). Data are mean with 95% confidence interval (C.I.) (EC_50_) or mean ± S.E.M. (*E*_max_), n = 6 independent experiments performed in triplicate. Statistical analyses were by non-overlapping C.I. or two-way ANOVA followed by Bonferroni’s post-hoc test. *p < 0.05 relative to CP55,940 within assay. n. d, not determined; n. c, not converged. *E*_max_ values for data that were not fit to a non-linear regression are the mean from the maximum value observed.

Among the compounds tested, **JR64a** and **JR14a**, were the most interesting of series **A** and **B** respectively (Table 1; Figure 2B). In particular **JR64a** was more potent than the orthosteric agonist **LV62** (EC_50(JR64a)_ = 8.6 nM; EC_50(LV62)_ = 58 nM [not statistically significant]); and its potency was also almost comparable to that observed in **EC21a** and **LV62** co-administration experiments (EC_50(LV62+EC21a)_ = 4.3 nM). All other tested **JR** compounds displayed lower potency compared to the parent orthosteric agonist **LV62** or the compounds **JR64a** (Table 1). Only a modest efficacy, ranging from 38 to 41%, and lower than that observed in **EC21a** and **LV62** co-administration experiments (E_max(LV62+EC21a)_ = 104 ± 5.2), was observed for both **JR14a** and **JR64a** (Table 1). Therefore, the combination of **EC21a** (PAM) and **LV62** (agonist) produced greater potency and efficacy than the resultant **JR** compounds, obtained conjoining these two ligands. Nevertheless, our results indicated that the ligands **JR14a** and **JR64a** displayed modest partial agonist activity in the inhibition of FSK-stimulated cAMP accumulation assay in *h*CB_2_R-expressing cells.

**FIGURE 3:**
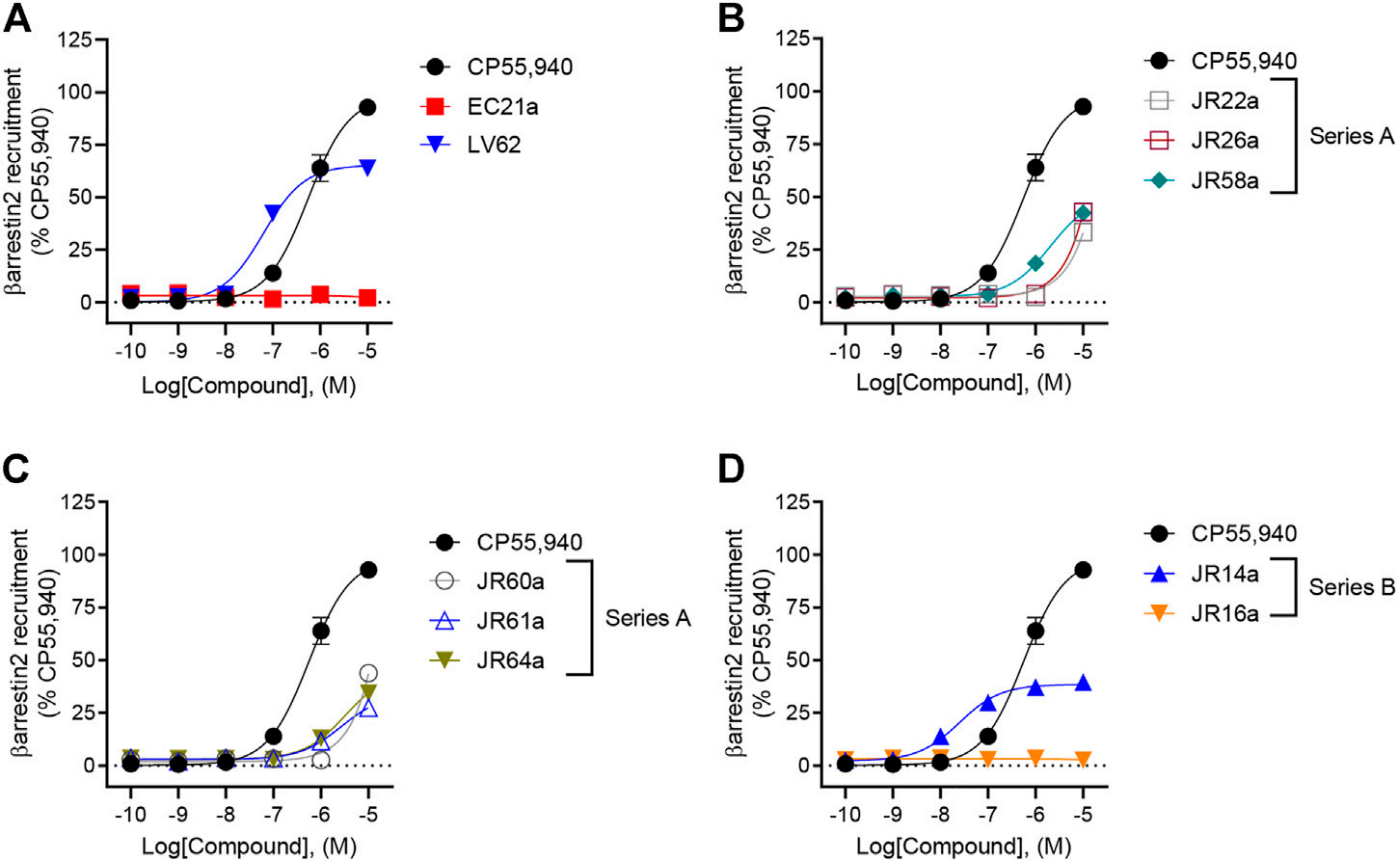
Evaluation of *h*CB_2_R-dependent recruitment of βarrestin2. CHO-K1 cells stably-expressing *h*CB_2_R were treated with 0.10 nM—10 μM compounds for 90 min and βarrestin2 recruitment was measured. **(A)** Parent compounds used for the design of the JR derivatives; **(B,C)** compounds of series **A**; **(D)** compounds of series **B**. CP55,940 data are the same in all four panels and presented for reference. Data are expressed as % CP55,940 response. Data were fitted to a non-linear regression (3 parameter model, GraphPad v. 9.0). Data are mean ± S.E.M. of six independent experiments performed in triplicate.

In addition to G protein-mediated signaling, GPCRs also interact with βarrestins, which facilitates receptor internalization, recycling, degradation, and signaling (Chen et al., 2014; Donthamsetti et al., 2020). Therefore, the complete panel of new orthosteric/allosteric hybrid CB_2_R ligands and their corresponding parent compounds were evaluated for their ability to enhance βarrestin2 recruitment in CHO cells stably-expressing *h*CB_2_R. Cells were treated with CP55,490 or compound for 90 min (Figure 3; Table 1). Regarding the parent compounds, no response was detected for **EC21a** alone, which is consistent with its activity as a CB_2_R PAM (Figure 3A; Table 1). The CB_2_R orthosteric agonist **LV62** enhanced βarrestin2 recruitment with both potency and efficacy comparable to those previously observed in FSK-stimulated cAMP accumulation assays (Table 1). Interestingly, analogs **JR14a** and **JR64a**, previously shown to inhibit FSK-stimulated cAMP accumulation, exhibited different behaviors in enhancing βarrestin2 recruitment (Figure 3; Table 1). **JR14a** enhanced βarrestin2 recruitment with nanomolar potency (EC_50(JR14a)_ = 25 nM), whereas **JR64a**, did not significantly enhance βarrestin2 recruitment at concentrations below 10,000 nM. Therefore, **JR64a** displayed greater potency in the inhibition of cAMP accumulation as compared to the recruitment of βarrestin2 (biased ligand). This result may be important for the design of new CB_2_R agonists devoid of the side effects related to the internalization and desensitization of CB_2_Rs. Indeed, it has been reported that CB_2_R agonists, by enhancing βarrestin2 recruitment, can induce internalization and desensitization of the receptor leading to a decrease in signaling and surface receptor levels (Chen et al., 2014).

### [^3^H]CP55,940 binding assays

**FIGURE 4:**
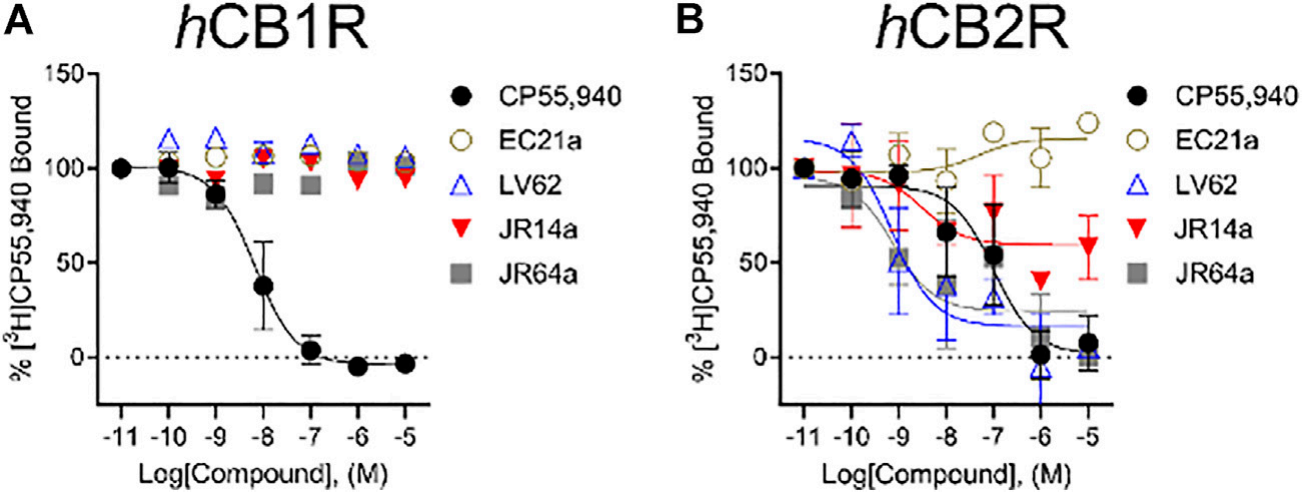
[^3^H]CP55,940 binding to *h*CB_1_R and *h*CB_2_R. Membranes from CHO-K1 cells stably-expressing *h*CB_1_R **(A)** or *h*CB_2_R **(B)** were incubated with 1 nM [^3^H]CP55,940 and 0.10 nM—10 μM compounds for 2 h. Data are expressed as % [^3^H]CP55,940 bound. Data were fitted to a non-linear regression (3 parameter model to estimate changes in displacement [IC_50_] which was substituted for EC_50_, GraphPad v. 9.0). Data are mean ± S.E.M. of at least three independent experiments performed in duplicate. Data from these graphs is presented in Table 2.

Following characterization of G protein-mediated cAMP inhibition and βarrestin2 recruitment, we assessed ligand affinity for **LV62**, **EC21a**, **JR14a**, and **JR64a** at *h*CB_1_R and *h*CB_2_R using a [^3^H]CP55,940 radioligand displacement assay on membranes derived from CHO-K1 cells stably-expressing either receptor. No affinity at *h*CB_1_R was detected for all compounds (Figure 4A; Table 2). At *h*CB_2_R*,* consistent with its CB_2_R PAM character, **EC21a** increased [^3^H]CP55,940 bound to *h*CB_2_R (Figure 4B; Table 2). **LV62** fully displaced [^3^H]CP55,940 from *h*CB_2_R, as expected for an orthosteric compound (Figure 4B; Table 2). Remarkably, novel *h*CB_2_R ligands **JR14a** and **JR64a** displayed nanomolar relative affinities in the [^3^H]CP55,940 displacement assay with *h*CB_2_R CHO-K1 cell membranes (Figure 4B; Table 2; IC_50(JR14a)_ = 1.8 nM; IC_50(JR64a)_ = 0.60 nM).

**TABLE 2:** [^3^H] CP55,940 binding.

Compound(s)	*h*CB1R		*h*CB2R	
	IC_50_ (nM) (95% C.I.)	*E*_min_ (% CP55,940) ± S.E.M	IC_50_ (nM) (95% C.I.)	*E*_min_ (% CP55,940) ± S.E.M
CP55,940	6.6 (2.7–15)	0.0 ± 5.6	40 (7.7–220)	1.5 ± 14.3
EC21a	n.c	103 ± 2.5***	36 (4.6–77)	115 ± 7.9***
LV62	n.c	106 ± 3.6***	0.65 (0.13–34)	16 ± 11.9
JR14a	n.c	98 ± 2.5***	1.8 (0.26–6.2)	59 ± 12.3*
JR64a	n.c	104 ± 4.7***	0.60 (0.20–26)	20 ± 10.3

Competition binding of [^3^H]CP55,940 to *h*CB_1_R and *h*CB_2_R was quantified in membranes derived from CHO-K1 *h*CB1R or *h*CB2R cells incubated with compounds for 2 h. Data were fit to a three-parameter non-linear regression in GraphPad (v. 9.0). Data are mean with 95% C.I. (IC_50_) or mean ± S.E.M, *n* ≥ 3 independent experiments performed in duplicate. Statistical analyses were by non-overlapping C.I. or two-way ANOVA followed by Bonferroni’s post-hoc test. *p < 0.05, ***p < 0.001 relative to CP55,940 within receptor. Data from this Table are graphed in Figure 4 n. c, not converged. *E*_min_ values for data that were not fit to a non-linear regression are the mean from the minimum value observed.

### Anti-inflammatory properties of selected CB_2_R ligands in human microglial cells (HMC3)

**FIGURE 5:**
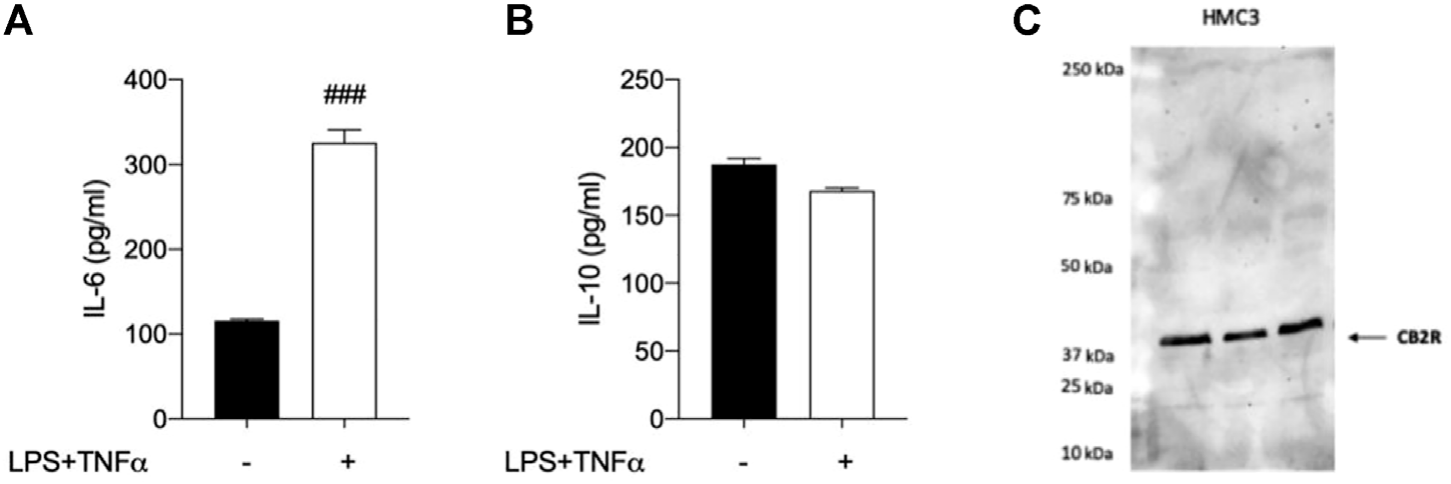
Analysis of HMC3 cell line response to inflammatory stimulus and expression of CB_2_R. **(A)** Pro-inflammatory interleukin-6 (IL-6) and **(B)** anti-inflammatory interleukin-10 (IL-10) levels were measured after exposure to LPS (10 μg/ml)/TNFα (50 ng/ml) stimulus for 24 h. **(C)** Western Blot (WB) analysis allowed the detection of CB2R in control HMC3 cell lysates. [A-B]: data represent means ± S.E.M. from *n* = 3 independent experiments, performed in duplicate. Statistical analysis was performed by ordinary one-way ANOVA followed by Tukey’s multiple comparison test. ###*p* < 0.005 compared to control cells.

**FIGURE 6:**
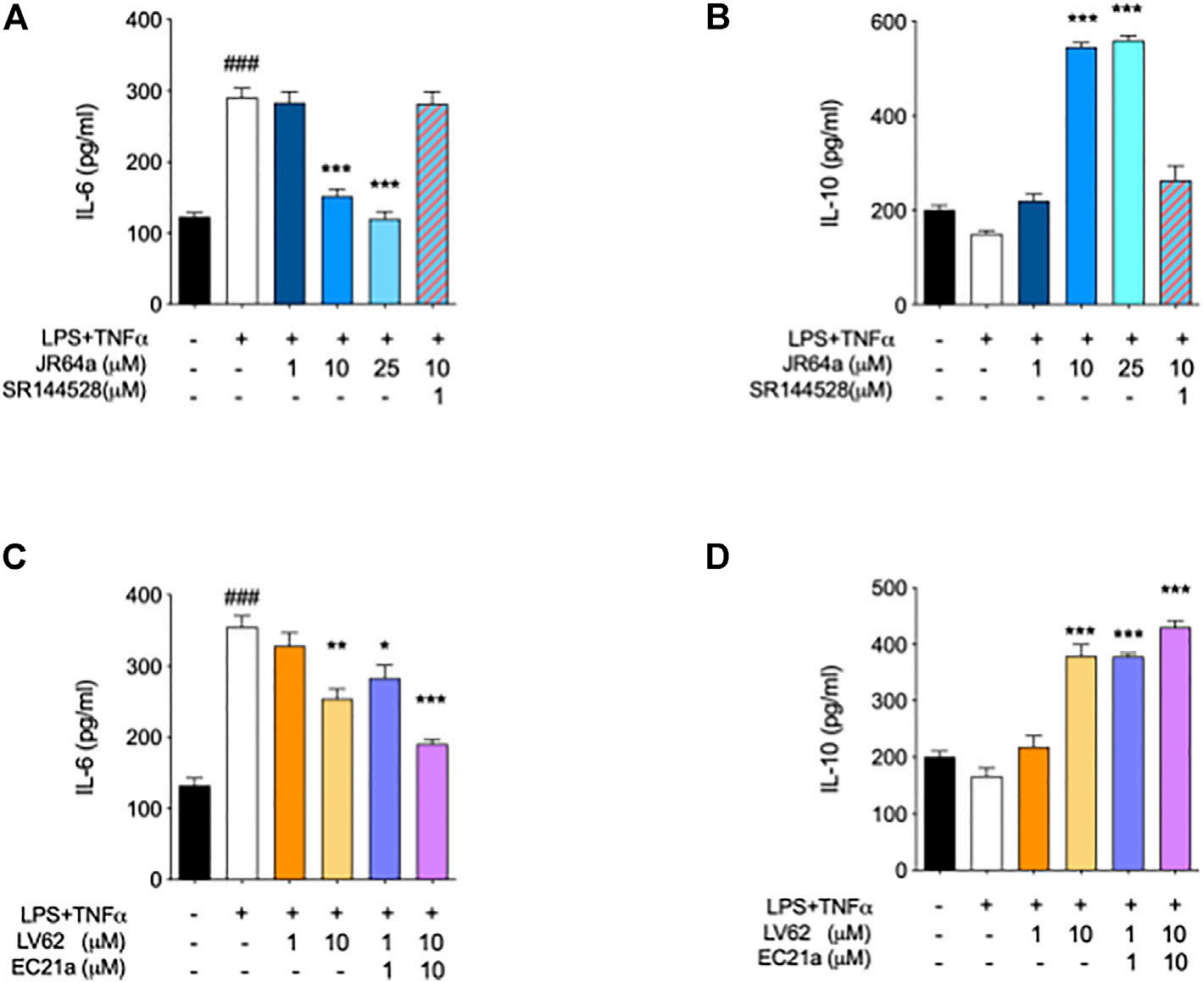
Ability of dualsteric CB_2_R ligand **JR64a** to decrease the inflammatory phenotype of LPS/TNFα stimulated HMC3 cells. Panels **(A)** and **(B)** show the effects of **JR64a** on the release of pro-and anti-inflammatory interleukines IL-6 and IL-10, respectively. Panels **(C)** and **(D)** show the effects produced in the same experimental setting by administering CB_2_R selective orthosteric agonist **LV62** alone or in combination (1:1) with the CB_2_R PAM **EC21a**. Bars represent the release (pg/ml) of ILs in the presence of the compounds. Data represent mean ± SEM from *n* = 3 independent experiments performed in duplicate. Statistical analysis was performed by ordinary one-way ANOVA followed by Tukey’s multiple comparison test. ^###^ *p* < 0.005 vs. control cells; *p < 0.05, **p < 0.01 and ***p < 0.005 vs. LPS/TNFα treated cells.

**FIGURE 7:**
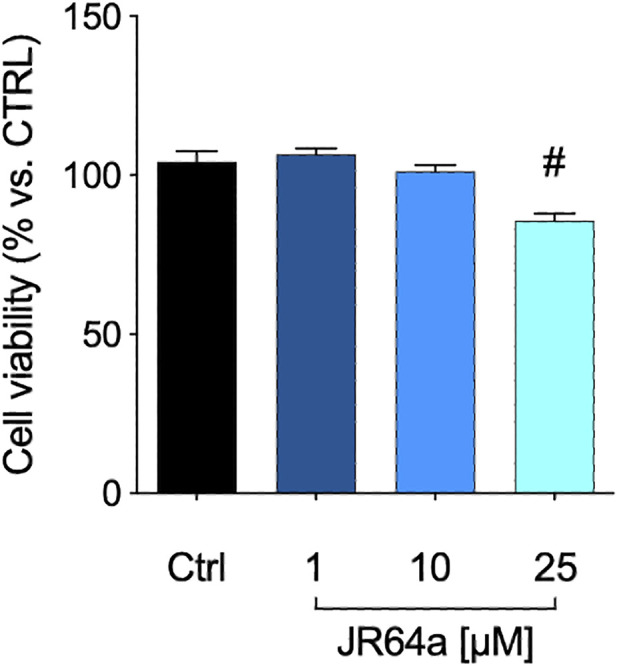
MTT assay performed with different concentrations of **JR64a**. Data represent means ± S.E.M. from *n* = 3 independent experiments, performed in triplicate. Statistical analysis was performed by ordinary one-way ANOVA followed by Tukey’s multiple comparison test. ^#^ *p* < 0.05 vs. control.

**FIGURE 8:**
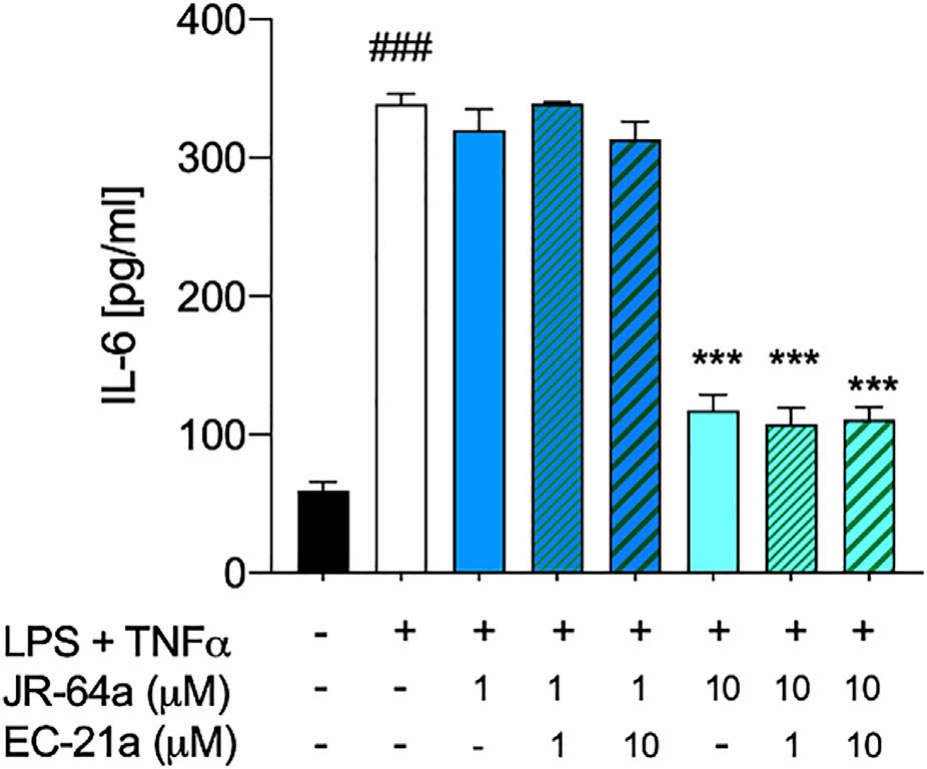
Co-administration of selective **CB**_**2**_ **R PAM EC21a** doesn’t modify the effects of **JR64a** on the release of IL-6 in HMC3 cells exposed to LPS/TNFα stimulus. Bars represent the release (pg/ml) of IL-6 in the presence of the compounds. Data represent mean ± SEM from *n* = 3 independent experiments performed in duplicate. Statistical analysis was performed by ordinary one-way ANOVA followed by Tukey’s multiple comparison test. ^###^ *p* < 0.005 vs. control cells; ***p < 0.005 vs. LPS/TNFα treated cells.

Taking into account that the inflammatory process in microglial cells supports the onset and progression of several neurodegenerative and psychiatric disorders (Nakagawa and Chiba, 2014; Hansen et al., 2018; Haukedal and Freude 2019; Ho 2019), we decided to investigate the anti-inflammatory properties of novel CB_2_R ligand **JR64a**, which had emerged from a preliminary screening as the most promising of the series. At this aim, we first set up a human model of microglial inflammation, by exposing HMC3 cells to LPS/TNFα stimulus (Dello Russo et al., 2018). In agreement with our previous observations (Polini et al., 2020; Gado et al., 2022), exposure of microglial cells to LPS/TNFα resulted in a significant increase of the release of pro-inflammatory IL-6 as compared to control cells (Figure 5A), whereas no significant effects were observed on IL-10 release (Figure 5B). Expression of CB_2_R in HMC3 microglial cells was also assessed by WB analysis (Figure 5C). Then, dose-response experiments were carried out by exposing HMC3 cells to pretreatment with increasing concentrations (1, 10, and 25 μM) of test compound followed by LPS/TNFα treatment for 24 h (Figure 6). Notably, no relevant cytotoxic effects were detected in HMC3 cells after treatment with **JR64a** at 1 and 10 μM concentrations (Figure 7), whereas when used at 25 μM a modest decrease in cell viability (ca 10%) was observed (Figure 7). Measurements of pro- and anti-inflammatory interleukins levels (i.e. IL-6 and IL-10) in cell media by ELISA tests revealed that, when used at 10 or 25 μM concentration, analog **JR64a** displayed a marked anti-inflammatory activity, which was completely abolished after pretreatment with the CB_2_R selective antagonist **SR144528** (1 μM, Figures 6A,B), suggesting a CB_2_R-mediated anti-inflammatory effect in HMC3 microglial cells. In parallel experiments, the anti-inflammatory effects of the orthosteric agonist **LV62** administered at 1 or 10 μM concentration either individually or in equimolar combination with the CB_2_R-PAM **EC21a** were evaluated. As shown in Figures 6C,D, administration of **EC21a** at equimolar dosages enhanced **LV62** anti-inflammatory activity, which resulted in a similar response to that observed on administration of equimolar concentrations of the hybrid compound **JR64a** (Figures 6A,B). Notably, co-administration of **EC21a** at equimolar or higher doses revealed no effects on **JR64a** anti-inflammatory activity in HMC3 cells (Figure 8), suggesting a potential ability for **JR64a** to interact with both orthosteric and allosteric sites of CB_2_R. However, additional investigation is needed to confirm the potential bitopic pharmacology of **JR64a**. In particular, computational studies using our recently proposed model of CB_2_R in complex with bitopic ligands (Gado et al., 2022) could certainly help to clarify the binding mode of **JR64a** and its ability of occupying both orthosteric and allosteric site of the CB_2_R, as well as to facilitate the rational design of novel and more effective CB_2_R bitopic ligands.

## Conclusion

Recent progress made in the discovery and functional characterization of GPCRs ligands have enabled the design of bitopic/duasteric ligand as single chemical entities in which an orthosteric and an allosteric pharmacophore are chemically attached via a linker in a manner to allow simultaneous targeting of two binding sites (orthosteric and allosteric) within one receptor. This approach may offer several advantages over the classical ‘monovalent’ ligand, such as an increased affinity and selectivity for the target receptor, often accompanied with functional selectivity (stimulus bias) (Newman et al., 2020). However, the design of bitopic ligands needs deep knowledge of orthosteric and allosteric ligands for a given GPCR as well as its binding sites (orthosteric and allosteric.). For these reasons, bitopic ligands have so far been described predominantly for muscarinic receptors (Valant, et al., 2008; Keov, et al., 2014), adenosine receptors (Valant et al., 2014), and dopamine receptors (Draper-Joyce et al., 2018), for which detailed knowledge on allosteric binding sites and ligands is available.

In this study we have presented the synthesis and functional characterization of novel potential CB_2_R dualsteric agents, designed as hybrid compounds composed of two distinct pharmacophoric units, namely the CB_2_R PAM **EC21a** and the CB_2_R orthosteric agonist **LV62,** linked through different alkyl chains. After a preliminary screening, which included Gα_i/o_ protein-dependent inhibition of FSK-stimulated cAMP accumulation, ligand induced recruitment of βarrestin2, and binding to *h*CB_2_R, analog **JR64a** was identified as the most promising dualsteric CB_2_R ligand. Indeed, a bias towards the modulation of the cAMP-pathway over the recruitment of βarrestin2 combined with high affinity for binding to *h*CB_2_R were observed. Subsequently, the lead compound was tested to evaluate its ability to decrease the inflammatory phenotype of LPS/TNFα-stimulated human microglial cells (HMC3). Our results indicated that analog **JR64a** displayed good anti-inflammatory properties when used at 10 μM concentration. Notably, as demonstrated by pharmacological antagonism, the anti-inflammatory effect displayed by **JR64a** revealed to be CB_2_R-mediated, and no relevant HMC3 cytotoxicity was also observed after administering **JR64a** at 10 μM concentration. Even though still at a preliminary level, functional assays indicated that the selective CB_2_R PAM analog **EC21a** was not able to increase the anti-inflammatory effects produced by **JR64a** in HMC3 cells, supporting the design of this compound as a potential CB_2_R dualsteric ligand. Certainly, the availability of dualsteric/bitopic ligands for the CB_2_R can provide a fundamental contribution to the clarification of the mechanism of action of this receptor. It is therefore essential to provide an unequivocal demonstration of a simultaneous allosteric/orthosteric mode of binding for new bitopic ligand candidates. The good news is that recent advances in CB_2_R structural biology might help in the construction of allosteric/bitopic ligands with a designed pharmacological profile. Therefore, future structural and functional studies will provide critical insights to confirm the actual CB_2_R-bitopic ligand behavior of our novel analog **JR64a**.

## Data Availability

The original contributions presented in the study are included in the article/Supplementary Material, further inquiries can be directed to the corresponding authors.
