# Supplementary material for: Design, synthesis and biological evaluation of novel orthosteric-allosteric ligands of the cannabinoid receptor type 2 (CB2R)
Source: Front Chem. 2022 Sep 27;10:984069. doi: 10.3389/fchem.2022.984069 (PMC9551276; doi:10.3389/fchem.2022.984069)

Supplementary Material

Ethyl 2-oxo-1,2-dihydro-1,8-naphthyridine-3-carboxylate (1). A solution of 2-aminopyridin-3-carboxaldehyde (1.00 g, 8.19 mmol) diethyl malonate (1.86 mL, 12.3 mmol) and few drops of piperidine (0.24 mL, 2.46 mmol) was heated at 125 °C for 20 h. After cooling down to room temperature, the solvent was quickly evaporated until a solid residue was obtained. The residue was filtered *in vacuum* condition and washed by using diethyl ether. The filtered solid was concentrated *in vacuo* to afford the compound **2** as a yellow solid and it was used in the next step without any further purification. The yield of the reaction is 89 % (1.59 g, 7.29 mmol). ^{1}H NMR ($CDCl_3$) δ 11.63 (bs, 1H, NH), 8.82 (dd, 1H, *J*=4.8 Hz; 1.8 Hz, H_7), 8.47 (s, 1H, H_4), 8.02 (dd, 1H, *J*=8.0 Hz, 1.8 Hz, H_5), 7.27 (m, 1H, H_6), 4.44 (q, 2H, *J*=7.2 Hz, CH_2), 1.43 (t, 3H, *J*= 7.2 Hz, CH_3).

1-(4-fluorobenzyl)-2-oxo-1,2-dihydro-1,8-naphthyridine-3-carboxylic acid (2). Cesium carbonate (6.65 g, 20.4 mmol) was added to a solution of **1** (1.59 g, 7.29 mmol) in dry *N*,*N*-dimethylformamide at room temperature. After 1 h, 4-fluorobenzyl bromide (1.75 mL, 14.6 mmol) was added, and the mixture was stirred at 50°C for 12 h. After removing the solvent, an alkaline hydrolysis of ester group in the presence of NaOH al 10% at reflux for 24 h was performed. After cooling, the reaction mixture was extracted with chloroform and water and the aqueous phase was isolated and acidified with HCl until pH 2−3. The precipitate formed was filtered *in vacuum* condition, treated with water, and dried to give the acid derivative **2** as white solid. The yield of the reaction is 60 % (1.30 g, 4.36 mmol). ^{1}H NMR ($CDCl_3$) δ 8.90 (s, 1H, H_4), 8.85 (dd, 1H, *J*=4.6 Hz; 1.6 Hz, H_7), 8.16 (dd, 1H, *J*=7.6 Hz, 1.6 Hz, H_5), 7.42 (dd, 1H, *J*=7.6 Hz, 4.6 Hz, H_6), 7.55 and 6.97 (AA'XX' system, 4H, Ar-H), 5.82 (s, 2H, CH_2).

***tert*-butyl(4-(1-(4-fluorobenzyl)-2-oxo-1,2-dihydro-1,8-naphthyridine-3-carboxamido)cyclohexyl)carbamate (3).** The derivative **2** (798 mg, 2.68 mmol) was dissolved in dry *N*,*N*-dimethylformamide and TBTU (1.03 g, 3.22 mmol) and NEt_3 (1.01 mL, 7.50 mmol) were added. The reaction mixture was stirred at 0°C for 30 min after which the *cis*-4-(Boc-amino) cyclohexylamine (574 mg, 2.68 mmol) was added. The reaction mixture was stirred at 0°C for 30 min and then at room temperature for 12 h. The solvent was evaporated, and the crude product partitioned between water and chloroform. The organic layer was dried over sodium sulfate, evaporated and the crude product was purified via flash column chromatography (n-hexane/ethyl acetate 3:7) to afford compound **3** (1.04 g, 2.10 mmol) as white solid. Yield: 78%. ^{1}H NMR ($CDCl_3$) δ 9.90 (bd, 1H, *J*=7.6Hz, NH), 8.89 (s, 1H, H_4), 8.72 (dd, 1H, *J*=4.6 Hz; 1.6 Hz, H_7), 8.09 (dd, 1H, *J*=7.6 Hz; 1.6 Hz, H_5), 7.48 (AA'XX' system, 2H, Ar-H), 7.30 (dd, 1H, *J*=7.6 Hz; 4.6 Hz, H_6), 6.97 (AA'XX' system, 2H, Ar-H), 5.78 (s, 2H, CH_2), 4.61 (m, 1H, CHN), 4.16 (bs, 1H, NHCOO), 3.64 (m, 1H, CHN), 1.81 (m, 6H, 3 x CH_2), 1.63 (m, 2H, CH_2), 1.45 (s, 9H, 3 x CH_3).

N-(4-aminocyclohexyl)-1-(4-fluorobenzyl)-2-oxo-1,2-dihydro-1,8-naphthyridine-3-carboxami-de (4). Trifluoroacetic acid (1.82 mL) was added dropwise to a solution of derivative **3** (450 mg, 0.91 mmol) in dichloromethane at -20°C. Reaction contents were allowed to stir at room temperature for 3 h. The solution was evaporated to dryness, the residual material was filtered *in vacuum* condition and washed by using diethyl ether. The obtained yellow solid was treated with NaOH al 10%, filtered under *vacuum*, and dried to give the amine derivative **4** (310 mg, 0.79 mmol) as whitish solid. Yield: 87%. ^{1}H NMR ($CDCl_3$) δ 9.95 (bd, 1H, *J*=7.6 Hz, NH), 8.88 (s, 1H, H_4), 8.72 (dd, 1H, *J*=4.6

Hz; 1.6 Hz, H_7), 8.08 (dd, 1H, *J*=7.6 Hz; 1.6 Hz, H_5), 7.49 (AA'XX' system, 2H, Ar-H), 7.29 (dd, 1H, *J*=7.6 Hz; 4.6 Hz, H_6), 6.97 (AA'XX' system, 2H, Ar-H), 5.78 (s, 2H, CH_2), 4.21 (m, 1H, CHN), 2.90 (m, 1H, CHN), 1.89 (m, 2H, CH_2), 1.75 (m, 4H, 2 x CH_2), 1.52 (m, 2H, CH_2).

N-(4-(5-azidopentanamido)cyclohexyl)-1-(4-fluorobenzyl)-2-oxo-1,2-dihydro-1,8-naphthyridi-ne-3-carboxamide (5). The overall reaction consisted of two steps. In the first step reaction, a sealed vial was charged with 5-bromovaleric acid (200 mg, 1.10 mmol), NaN_3 (215 mg, 3.31 mmol), and dry DMF. The resulting mixture was heated at 60 °C for 12 h. The mixture was allowed to cool to room temperature and concentrated under reduced pressure to obtain the 5-azidovaleric acid as a white solid that was used in the next step without further purification. In the second step, NEt_3 (0.21 mL, 1.57 mmol) and TBTU (215 mg, 0.67 mmol) were added to a solution of the freshly prepared 5-azidovaleric acid (80.2 mg, 0.56 mmol) in dry DMF. The resulting mixture was stirred at 0°C for 30 min and then the amino-derivative **4** (221 mg, 0.56 mmol) was added and left under stirring firstly at 0°C for 30 min and then at room temperature for 12 h. After this time, the solvent was evaporated and then the residue was diluted with water and extracted with $CHCl_3$. The organic layer was dried over Na_2SO_4, filtered, and concentrated to dryness. The crude product was purified with a flash column chromatography (n-hexane/ethyl acetate 1:9) and pure fractions containing the desired compound were evaporated to dryness affording the amide-derivative **5** as white solid (40.0 mg, 0.08 mmol). Yield: 14%. ^{1}H NMR ($CDCl_3$) δ 9.93 (bd, 1H, *J*=7.2 Hz, NH), 8.90 (s, 1H, H_4), 8.72 (dd, 1H, *J*=4.8 Hz; 2.0 Hz, H_7), 8.10 (dd, 1H, *J*=7.8 Hz; 2.0 Hz, H_5), 7.44 (AA'XX' system, 2H, Ar-H), 7.31 (dd, 1H, *J*=7.8 Hz; 4.8 Hz, H_6), 6.97 (AA'XX' system, 2H, Ar-H), 5.79 (s, 2H, CH_2), 5.54 (bd, 1H, *J*=8.4 Hz, NH), 4.21 (m, 1H, CHN), 3.96 (m, 1H, CHN), 3.31 (t, 2H, *J*=6.8 Hz, CH_2N_3), 2.21 (t, 2H, *J*=7.2 Hz, CH_2CONH), 1.70 (m, 12H, cyclohexyl + 2xCH_2).

General Procedure for the Synthesis of N1-Substituted *N*-(4-methylcyclohexyl)-2-oxo-1,2-dihydro-1,8-naphthyridine-3-carboxamides (7, 9-11)

To a solution of derivative **6** (500 mg, 1.75 mmol) in anhydrous DMF (5.3 mL) was added cesium fluoride (798 mg, 5.25 mmol) and the mixture was stirred at the room temperature for 1h. Then, a proper di-halogenated alkanes or di-halogenated aliphatic ether (5.25 mmol) was added and stirring was continued at 30°C for 24 h. After cooling, the solvent was removed under chemical hood at 70°C and then the residue was washed with water and extracted with $CHCl_3$. The combined organic phase was dried over anhydrous Na_2SO_4 and evaporated to dryness to give the crude product which was purified by flash column chromatography to obtain the corresponding N1-substituted derivatives **7, 9-11**.

***1-(*5-bromopentyl*)-N-*(4-methylcyclohexyl)-2-oxo-1,2-dihydro-1,8-naphthyridine-3-carboxamide (7)**.

Purified by flash chromatography on a silica gel using hexane/AcOEt, 5:5. Yield: 86%. ^{1}H-NMR ($CDCl_3$) δ 10.01 and 9.63 (2d, 1H, *J*= 7.2 Hz, NH); 8.87 (s, 1H, H_4); 8.72 (dd, 1H, *J*= 4.6 Hz, 2.0 Hz, H_7); 8.08 (dd, 1H, *J*= 7.6 Hz, 2.0 Hz, H_5); 7.28 (dd, 1H, *J*= 7.6 Hz, 4.6 Hz, H_6); 4.60 (2t, 2H, *J*= 7.8 Hz, NCH_2); 4.27 and 3.92 (2m, 1H, CH); 3.45 (2t, 2H, *J*= 6.8 Hz, CH_2Br); 1.60 (m, 15H, cyclohexyl + 3xCH_2); 0.99 and 0.92 (2d, 3H, *J*= 6.8 Hz, CH_3).

***1-(*7-bromoeptyl*)-N-*(4-methylcyclohexyl)-2-oxo-1,2-dihydro-1,8-naphthyridine-3-carboxam-de (9)**.

Purified by flash chromatography on a silica gel using hexane/AcOEt, 5:5. Yield: 70 %. ^{1}H-NMR ($CDCl_3$) δ 10.01 and 9.66 (2d, 1H, *J*= 7.6 Hz, NH); 8.86 (s, 1H, H_4); 8.70 (dd, 1H, *J*= 4.6 Hz, 1.8 Hz, H_7); 8.05 (dd, 1H, *J*= 7.8 Hz, 1.8 Hz, H_5); 7.25 (dd, 1H, *J*= 7.8 Hz, 4.6 Hz, H_6); 4.57 (2t, 2H, *J*= 7.8 Hz, NCH_2);

4.24 and 3.92 (2m, 1H, CH); 3.40 (2t, 2H, *J*= 6.8 Hz, CH_2Br); 1.57 (m, 19H, cyclohexyl + 5xCH_2); 0.97 and 0.90 (2d, 3H, *J*= 6.6 Hz, CH_3).

1-(2-(2-bromoethoxy)ethyl)-N-(4-methylcyclohexyl)-2-oxo-1,2-dihydro-1,8-naphthyridine-3-carboxamide (**10**).
Purified by flash chromatography on a silica gel using hexane/AcOEt, 5:5. Yield: 74 %. ^{1}H-NMR ($CDCl_3$) δ 9.98 and 9.59 (2m, 1H, NH); 8.87 (s, 1H, H_4); 8.70 (dd, 1H, *J*= 4.6 Hz, 1.4 Hz, H_7); 8.07 (dd, 1H, *J*= 7.8 Hz, 1.4 Hz, H_5); 7.28 (dd, 1H, *J*= 7.8 Hz, 4.6 Hz, H_6); 4.87 (2t, 2H, *J*= 6.2 Hz, NCH_2); 4.26 and 3.92 (2m, 1H, CH); 3.90 (2t, 2H, *J*= 6.2 Hz, CH_2Br); 3.83 (t, 2H, *J*= 6.2 Hz, OCH_2); 3.37 (t, 2H, *J*= 6.2 Hz, OCH_2); 1.57 (m, 9H, cyclohexyl); 0.97 and 0.92 (2d, 3H, *J*= 6.4 Hz, CH_3).

1-(4-(4-bromobutoxy)butyl)-N-(4-methylcyclohexyl)-2-oxo-1,2-dihydro-1,8-naphthyridine-3-carboxamide (**11**).
Purified by flash chromatography on a silica gel using hexane/AcOEt, 5:5. Yield: 68 %. ^{1}H-NMR ($CDCl_3$) δ 10.02 and 9.64 (2m, 1H, NH); 8.80 (s, 1H, H_4); 8.64 (dd, 1H, *J*= 4.6 Hz, 1.8 Hz, H_7); 8.01 (dd, 1H, *J*= 7.8 Hz, 1.8 Hz, H_5); 7.21 (dd, 1H, *J*= 7.8 Hz, 4.6 Hz, H_6); 4.56 (2t, 2H, *J*= 7.6 Hz, NCH_2); 4.25 and 3.90 (2m, 1H, CH); 3.49 (2t, 2H, *J*= 6.6 Hz, CH_2Cl); 3.41 (m, 4H, 2xOCH_2); 1.58 (m, 17H, cyclohexyl + 4xCH_2); 0.92 and 0.85 (2d, 3H, *J*= 6.6 Hz, CH_3).

***1-(*5-azidopentyl*)-N-*(4-methylcyclohexyl)-2-oxo-1,2-dihydro-1,8-naphthyridine-3-carboxami-de (8)**
A mixture of 1:1 *trans* and *cis* isomers of the bromo derivative **6** (0.46 mmol) in anhydrous DMF and NaN_3 (89.7 mg, 1.38 mmol) was heated in a sealed vial at 60°C for 12h. The reaction mixture was then cooled to room temperature, concentrated to remove the solvent, treated with water, and extracted with dichloromethane. The organic phase was dried over anhydrous sodium sulfate, filtered, and evaporated under reduced pressure. The crude mixture was purified by flash chromatography on a silica gel using hexane/AcOEt, 6:4. Yield 99%. ^{1}H-NMR ($CDCl_3$) δ 10.00 and 9.64 (2d, 1H, *J*= 7.6 Hz, NH); 8.86 (s, 1H, H_4); 8.70 (dd, 1H, *J*= 4.6 Hz, 1.8 Hz, H_7); 8.08 (dd, 1H, *J*= 7.8 Hz, 1.8 Hz, H_5); 7.28 (dd, 1H, *J*= 7.8 Hz, 4.6 Hz, H_6); 4.60 (2t, 2H, *J*= 7.6 Hz, NCH_2); 4.25 and 3.92 (2m, 1H, CH); 3.31 (2t, 2H, *J*= 6.8 Hz, CH_2N_3); 1.59 (m, 15H, cyclohexyl + 3xCH_2); 0.98 and 0.92 (2d, 3H, *J*= 6.4 Hz, CH_3).

3-amino-4-methylpyridin-2(1H)-one (12).
Iron powder (1.825 g, 32.63 mmol) and ammonium chloride (0.922 g, 17.23 mmol) were added to a solution of the commercially available 2-hydroxy-4-methyl-3-nitropyridine (0.400 g, 2.61 mmol) in ethanol (16 mL) and water (8 mL). The reaction mixture was refluxed for 3 h, filtered under vacuum using celite and evaporated under reduced pressure. The obtained residue was dissolved in CHCl3 and washed with water. The organic phase was dried over Na2SO4, filtered and evaporated under reduced pressure giving the desired compound 12 as a brown solid (0.294 g, 2.37 mmol), which was used in the next step without any further purification. Yield: 91%. 1H NMR (CDCl3) δ: 12.27 (bs, 1H, NH), 6.77 (d, 1H, J=6.8 Hz, H6-Py), 6.07 (d, 1H, J=6.8 Hz, H5-Py), 4.01 (bs, 2H, NH2), 2.09 (s, 3H, CH3-Py).

N-(1,2-dihydro-4-methyl-2-oxopyridin-3-yl)-cycloheptanecarboxamide (13).
Cycloheptane carboxylic acid (0.86 ml, 6.29 mmol) was dissolved in C2O2Cl2 (1.59 ml, 18.87 mmol) with 3 drops of DMF. The solution was stirred at room temperature for 0.5 h and then the excess of C2O2Cl2 was removed by evaporation under nitrogen flux. The obtained acyl chloride was added dropwise to a solution of 12 (0.585 g, 4.71 mmol) and triethylamine (3.18 ml, 23.56 mmol) in

anhydrous DMF at 0 °C. The reaction mixture was stirred at room temperature for 24 h and then the solvent was removed under reduced pressure to give a residue which was dissolved in CHCl3 and washed three times with water. Subsequently the organic phase was dried over Na2SO4, filtered and evaporated under reduced pressure to afford a brown oil, which was triturated in methanol to obtain compound 13 as a white solid (0.621 g, 2.50 mmol). Yield: 53%. 1H NMR (CDCl3) δ: 12.26 (bs, 1H, NH), 7.51 (bs, 1H, NH), 7.14 (d, 1H, J=6.8 Hz, H6-Py), 6.27 (d, 1H, J=6.8 Hz, H5-Py), 2.53 (m, 1H, CHCO), 2.18 (s, 3H, CH3-Py), 2.02 (m, 2H, CH2), 1.78 (m, 4H, 2×CH2), 1.58 (m, 6H, 3×CH2).

N-(5-bromo-1,2-dihydro-4-methyl-2-oxopyridin-3-yl)-cycloheptanecarboxamide (14).
A solution of Br2 (0.14 mL, 2.73 mmol) in CHCl3 (1.81 mL) was added dropwise to a solution of derivative 8 (0.270 g, 1.09 mmol) in CHCl3. Reaction mixture was stirred at room temperature overnight and then was washed four times with a saturated aqueous solution of Na2S2O3. The organic phase was then dried over Na2SO4, filtered and evaporated under reduced pressure to afford a soid residue, which was triturated in ethyl acetate giving the desired compound 14 (0.330 g, 1.00 mmol) as a beige solid. Yield: 92%. 1H NMR (CDCl3) : 12.23 (bs, 1H, NH), 7.50 (bs, 1H, NH), 7.43 (s, 1H, H6-Py), 2.53 (m, 1H, CHCO), 2.23 (s, 3H, CH3-Py), 2.02 (m, 2H, CH2), 1.78 (m, 4H, 2×CH2), 1.54 (m, 6H, 3×CH2).

N-(5-bromo-1,2-dihydro-4-methyl-2-oxo-1-(pent-4-yn-1-yl)-pyridin-3yl)-cycloheptanecarboxamide (15).
Cesium fluoride (0.638 g, 4.20 mmol) was added to a solution of compound 14 (0.458 g, 1.40 mmol) in anhydrous DMF (4.20 mL). After 1 h at room temperature, 5-chloro-1-pentyne (0.44 mL, 4.20 mmol) was added, and the resulting mixture was left under stirring at 30 °C for 12 h. After that the solvent was removed under reduced pressure and the residue obtained was dissolved in CHCl3 and washed three times with water. The organic phase was dried over Na2SO4, filtered and evaporated under reduced pressure yielding a crude product which was purified by flash chromatography on silica gel using ethyl acetate/hexane 4:6 as eluent to afford compound 15 (0.416 g, 1.06 mmol) as white solid. Yield: 76%. Mp: 117-120°C. 1H-NMR: (CDCl3) δ (ppm) 7.54 (bs, 1H, NH), 7.38 (s, 1H, H6-Py), 4.03 (t, 2H, J=7.0 Hz, CH2NCO), 2.50 (m, 1H, CHCO), 2.25 (dt, 2H, J=6.8 Hz, J=2.6 Hz, CH2C), 2.15 (s, 3H, CH3-Py), 2.05 (t, 1H, J=2.6 Hz, CCH), 1.98 (m, 4H, 2xCH2), 1.75 (m, 4H, 2×CH2), 1.54 (m, 6H, 3×CH2).

N-(5-bromo-1,2-dihydro-4-methyl-2-oxo-1-(prop-2-yn-1-yl)-pyridin-3-yl)-cycloheptanecarboxamide (16).
Compound 16 was prepared from compound 14, as described for compound 15, using propargyl bromide and purified by flash column chromatography on silica gel using ethyl acetate/hexane 4:6 as eluent. Yield: 54 %. Mp: 175-178°C. 1H-NMR: (CDCl3) δ (ppm) 7.65 (bs, 1H, NH), 7.42 (s, 1H, H6-Py), 4.72 (d, 2H, J = 2.6 Hz, CH2NCO), 2.52 (t, 1H, J=2.6 Hz, CCH), 2.50 (m, 1H, CHCO), 2.17 (s, 3H, CH3-Py), 1.99 (m, 2H, CH2), 1.77 (m, 4H, 2×CH2), 1.56 (m, 6H, 3×CH2).

HPLC chromatogram of **JR14**

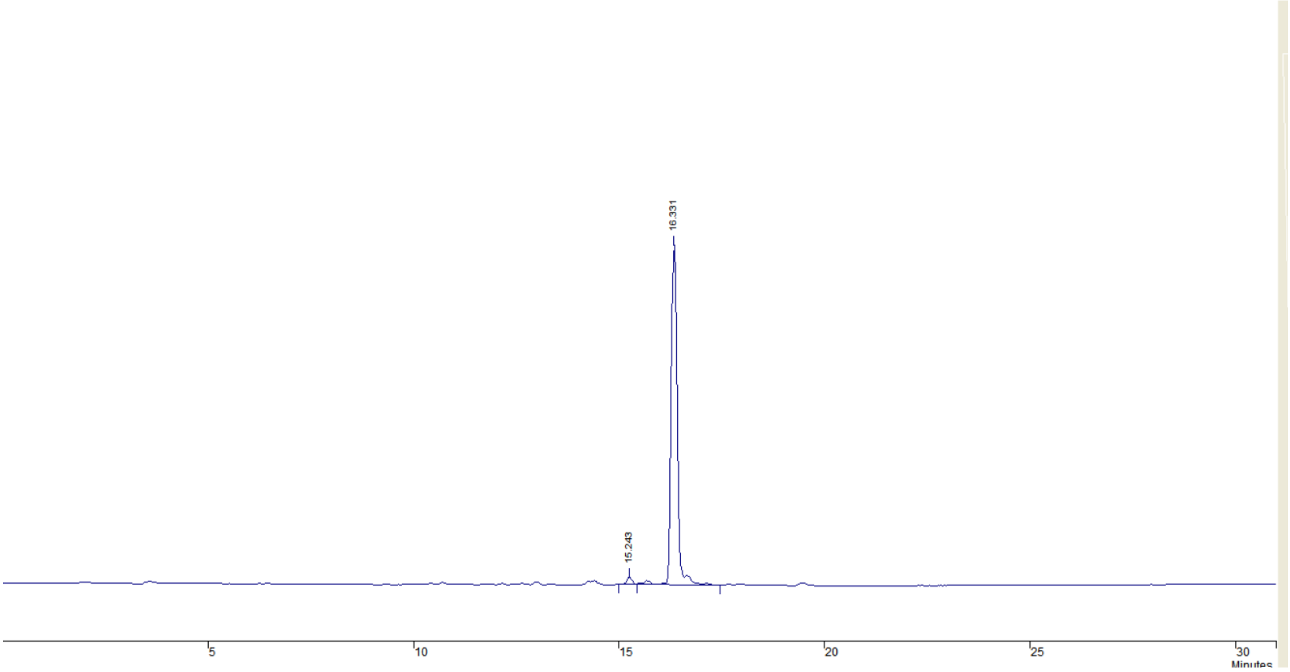

Peak No.	Peak Name	Result ()	Ret. Time (min)	Time Offset (min)	Area (counts)	Sep. Code	Width 1/2 (sec)	Status Codes
1		1.8472	15.243	0.000	973747	BV	8.0	
2		98.1528	16.331	0.000	51741992	VB	9.4	
	Totals:	100.0000		0.000	52715739			

HPLC chromatogram of **JR64**

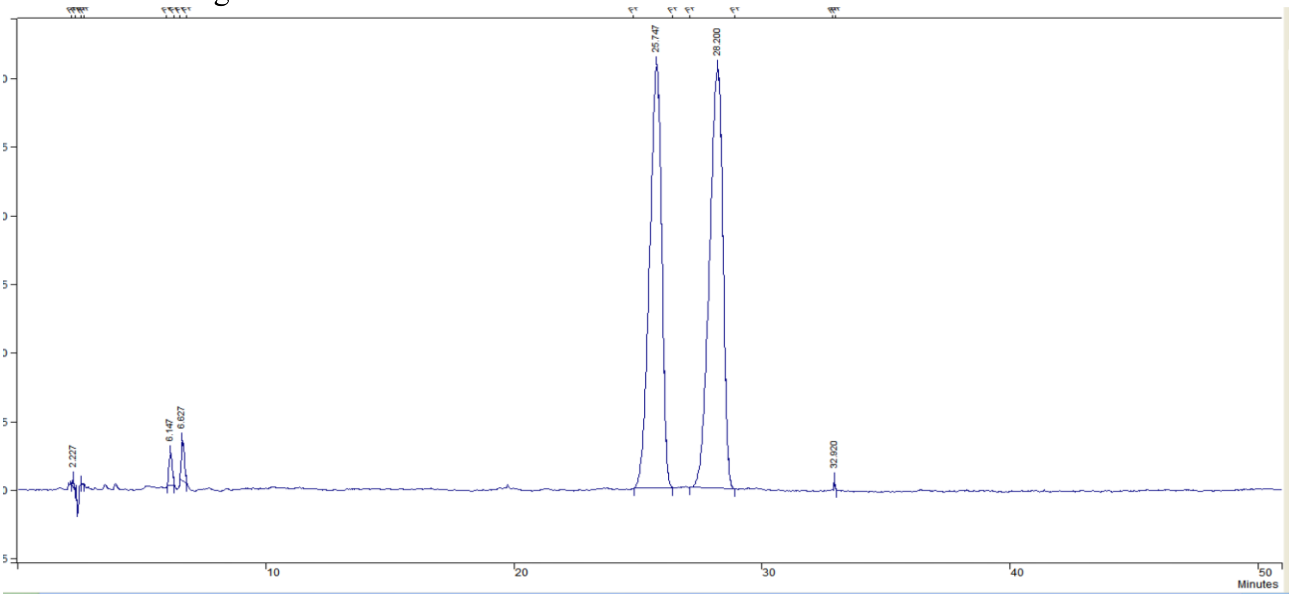

** LC Workstation Version 6.41 ** 01938-61c0-ea4-04b0 **

Run Mode : Analysis
Peak Measurement: Peak Area
Calculation Type: Percent

Peak No.	Peak Name	Result ()	Ret. Time (min)	Time Offset (min)	Area (counts)	Sep. Code	Width 1/2 (sec)	Status Codes
1		0.0797	2.227	0.000	45970	BB	2.7	
2		0.9351	6.147	0.000	539611	BB	8.8	
3		1.1497	6.627	0.000	663464	BB	8.4	
4		46.6175	25.747	0.000	26902252	BB	32.2	
5		51.2087	28.200	0.000	29551760	BB	35.8	
6		0.0095	32.920	0.000	5468	BB	1.1	
	Totals:	100.0002		0.000	57708525			

JR14a ^{1}H-NMR

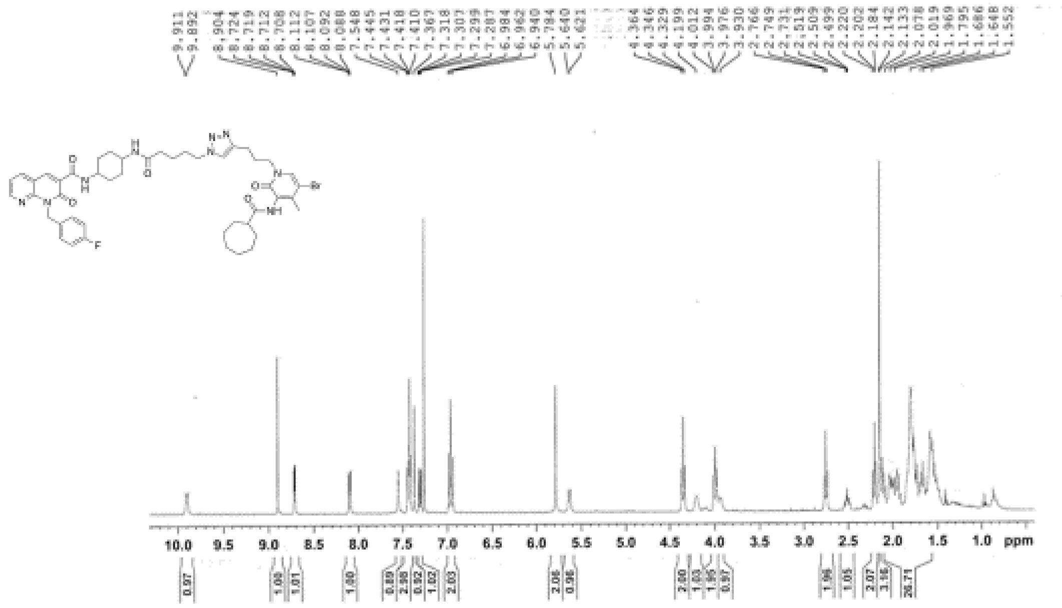

JR14a ^{13}C-NMR

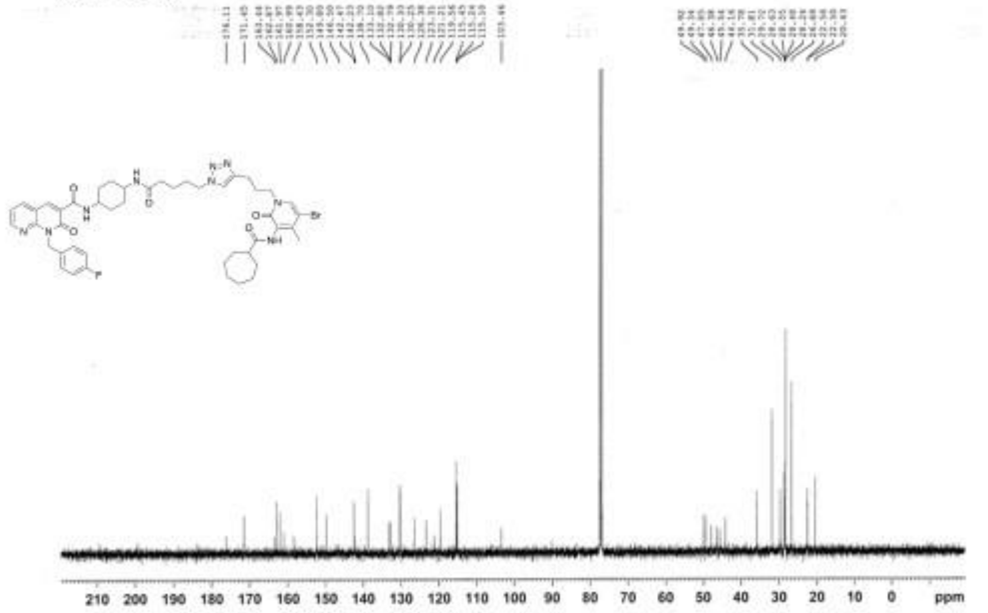

JR16a ^{1}H-NMR

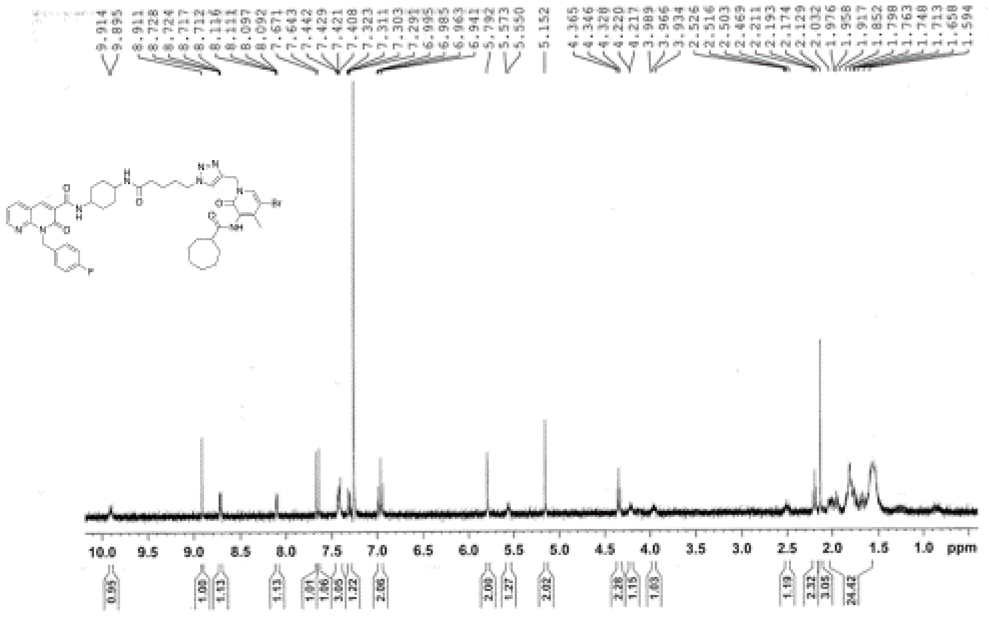

JR16a ^{13}C-NMR

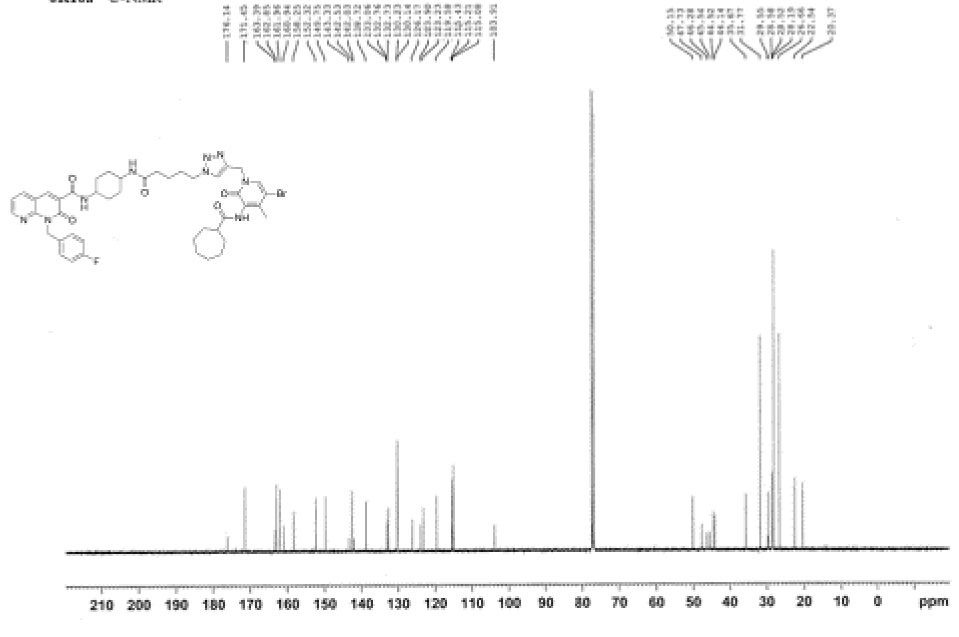

JR22a ¹H-NMR

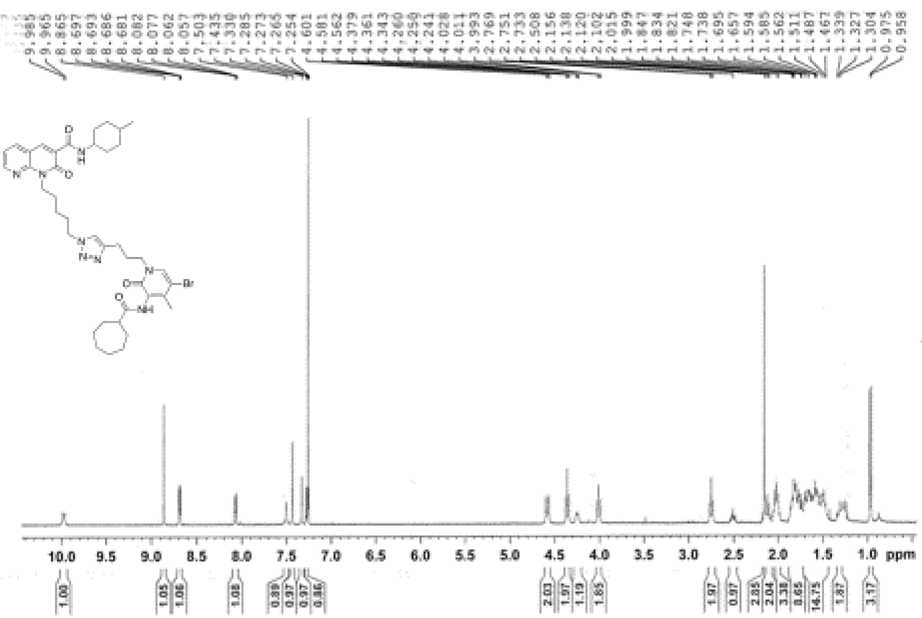

JR22a ¹³C-NMR

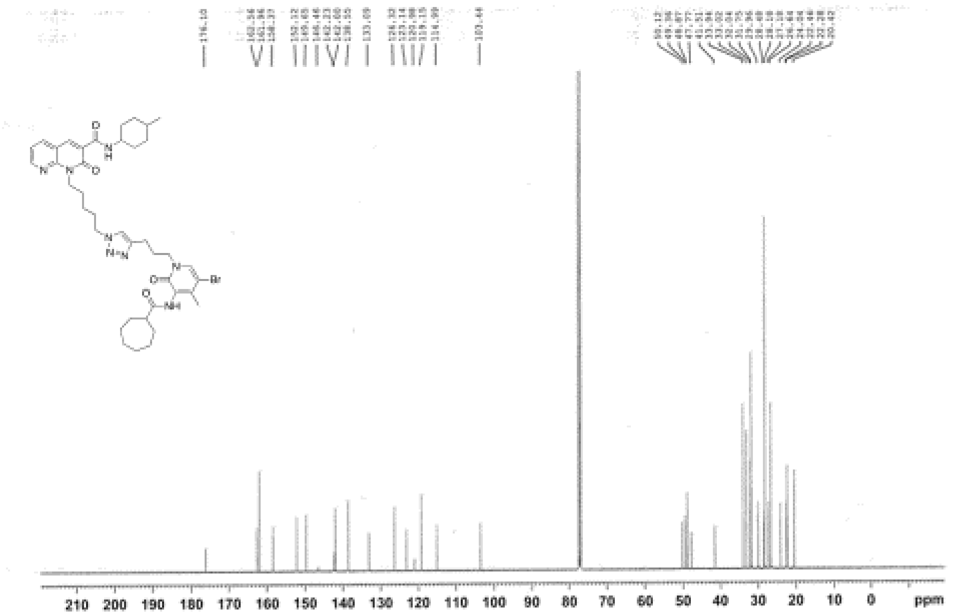

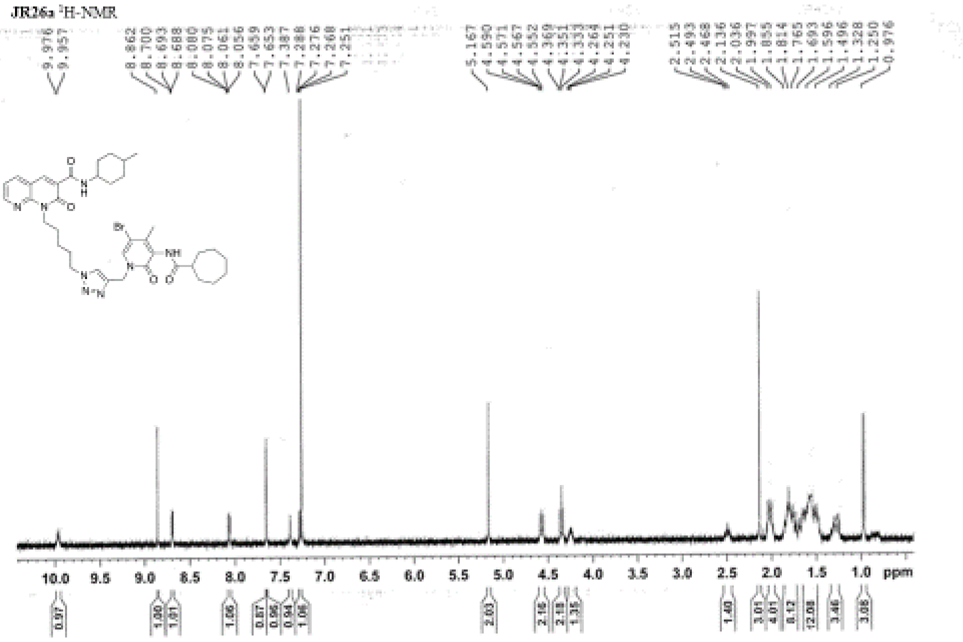
JR26a ¹H-NMR
ppm

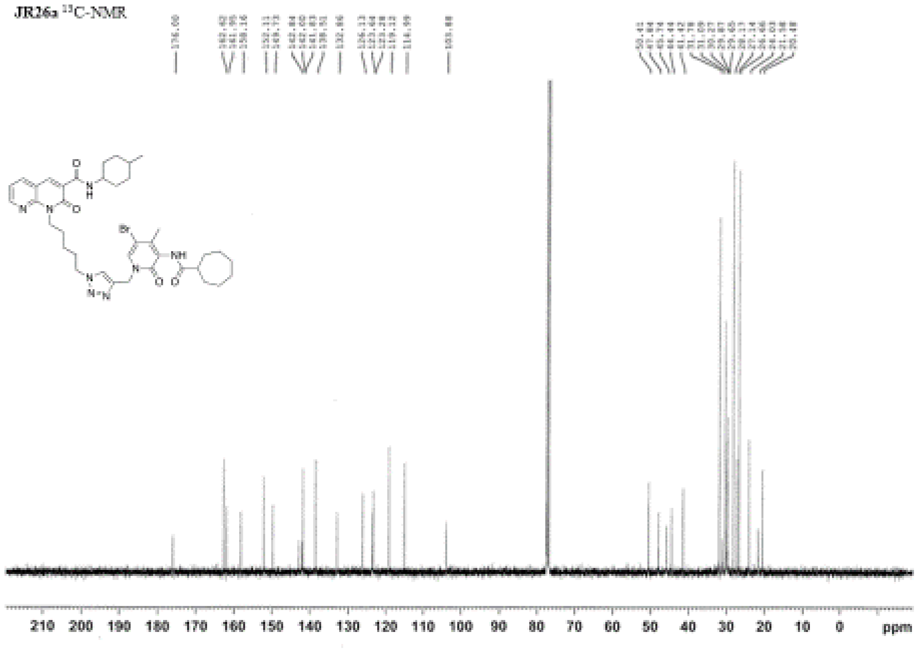
JR26a ¹³C-NMR
ppm

JR61a ^{1}H-NMR

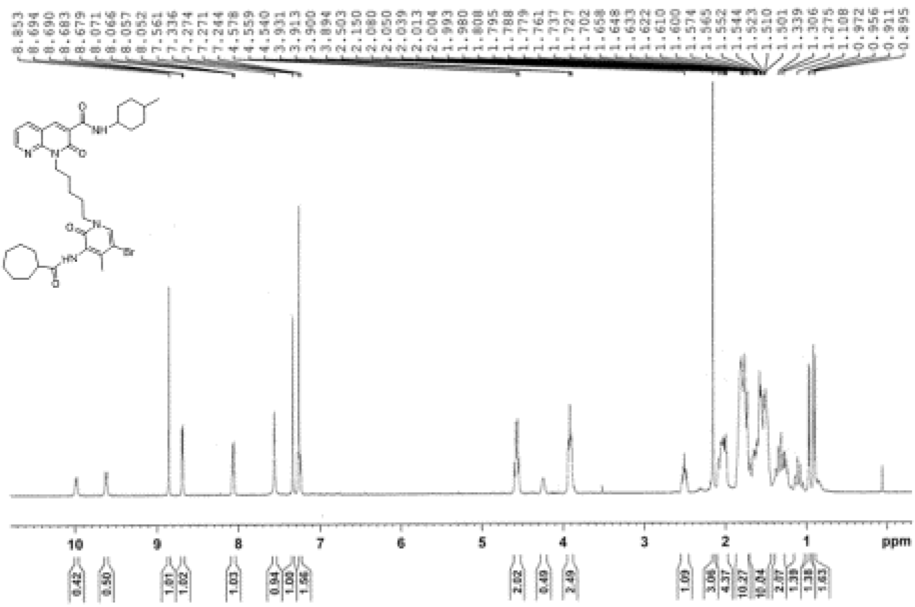

JR61a ^{13}C-NMR

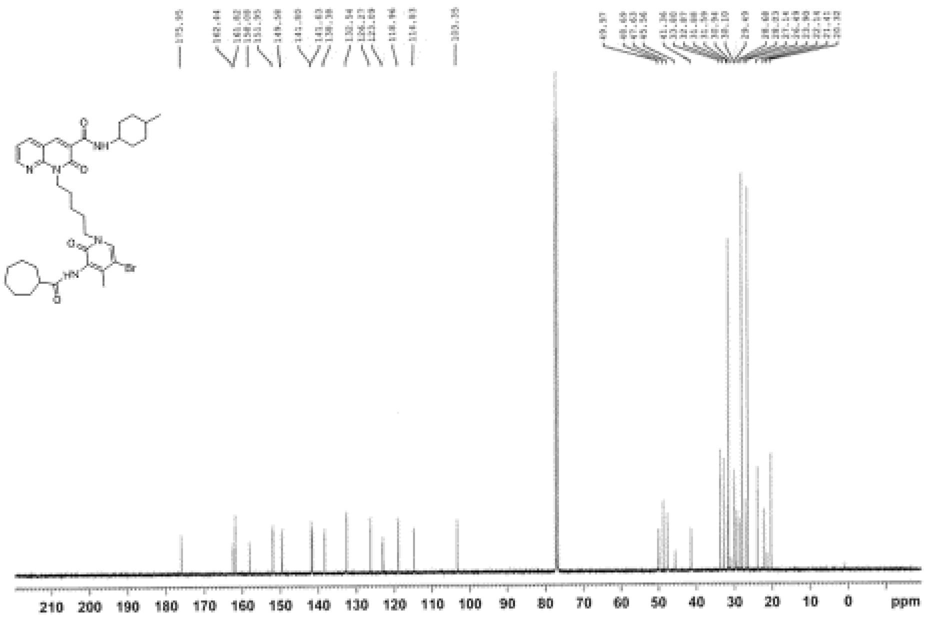

JR64a ^{1}H-NMR

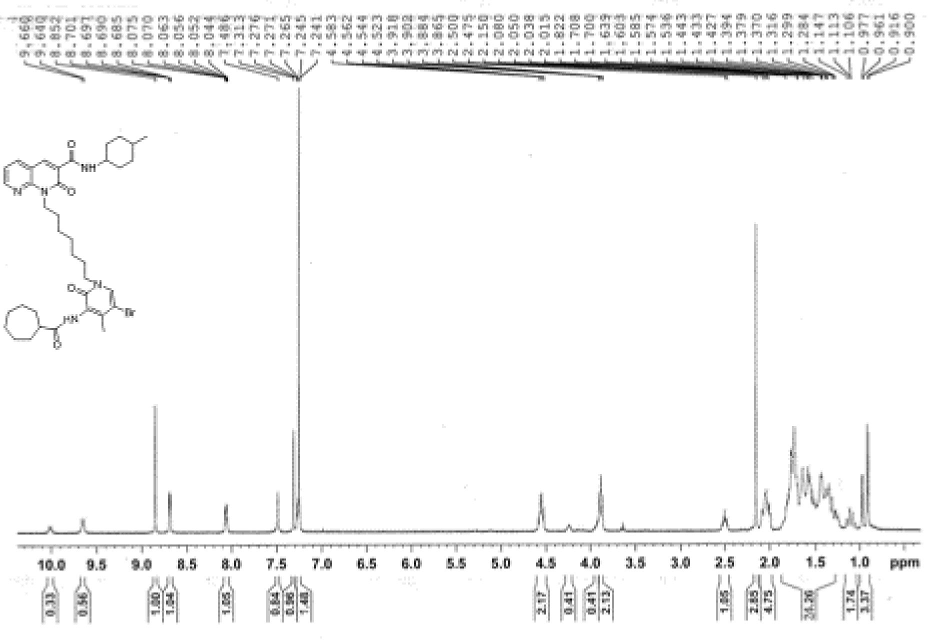

JR64a ^{13}C-NMR

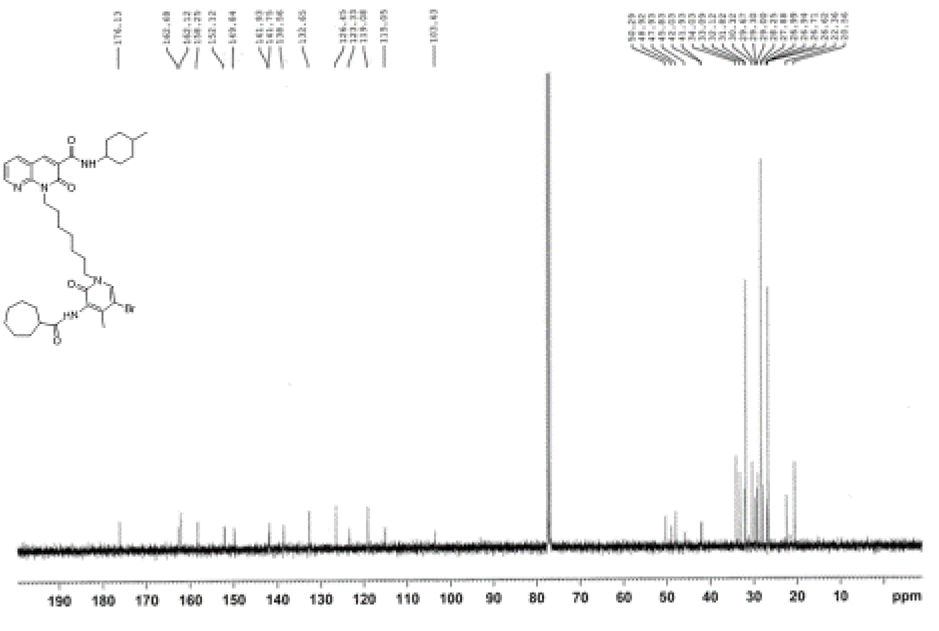

JR58a ^{1}H-NMR

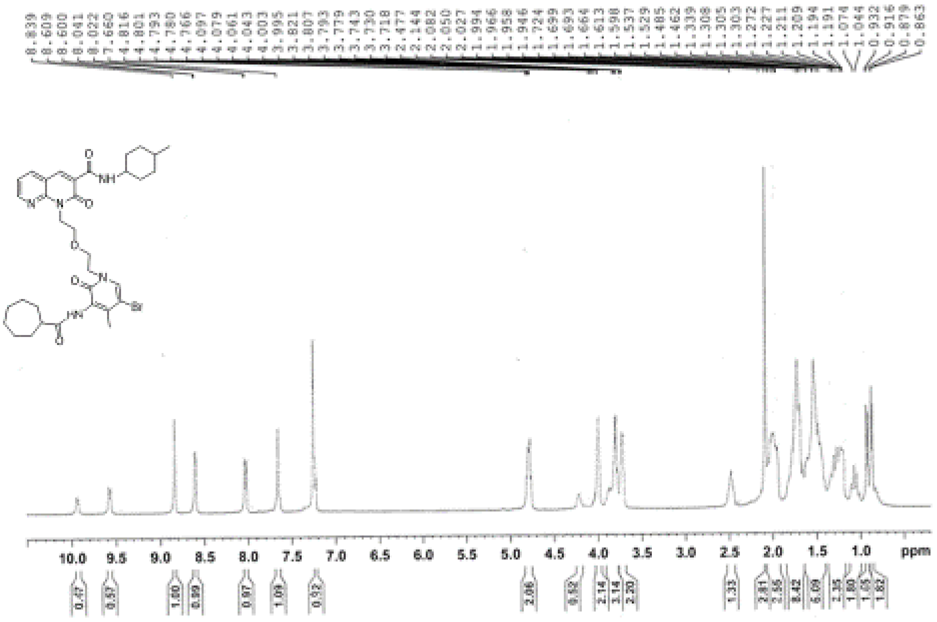

JR58a ^{13}C-NMR

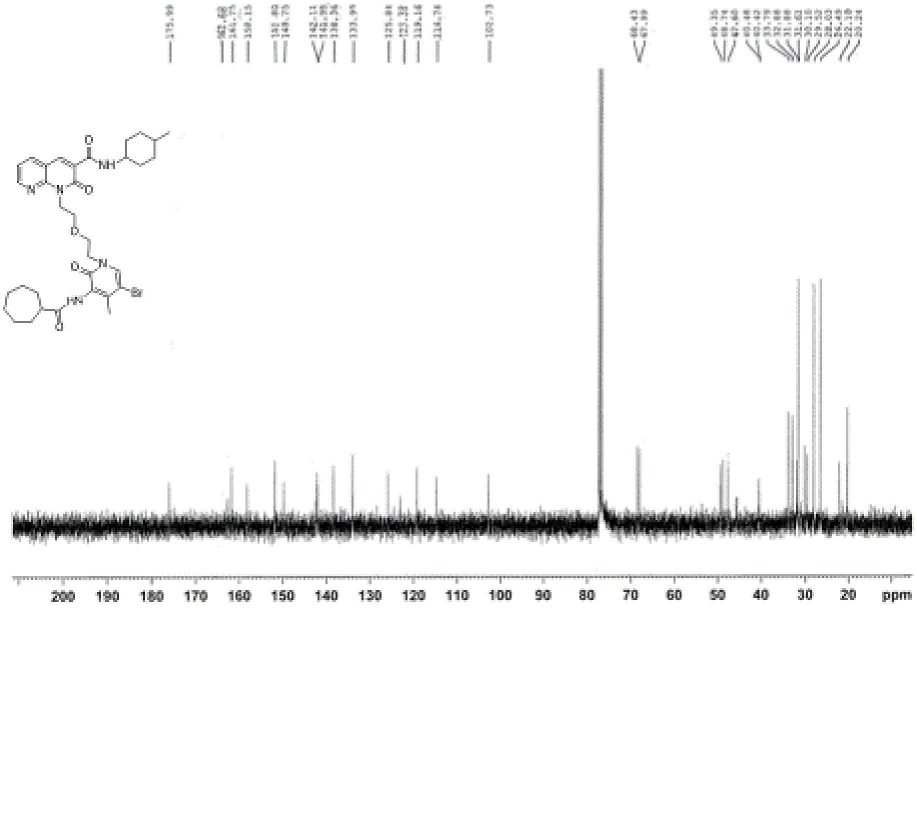

JR60a ^{1}H-NMR

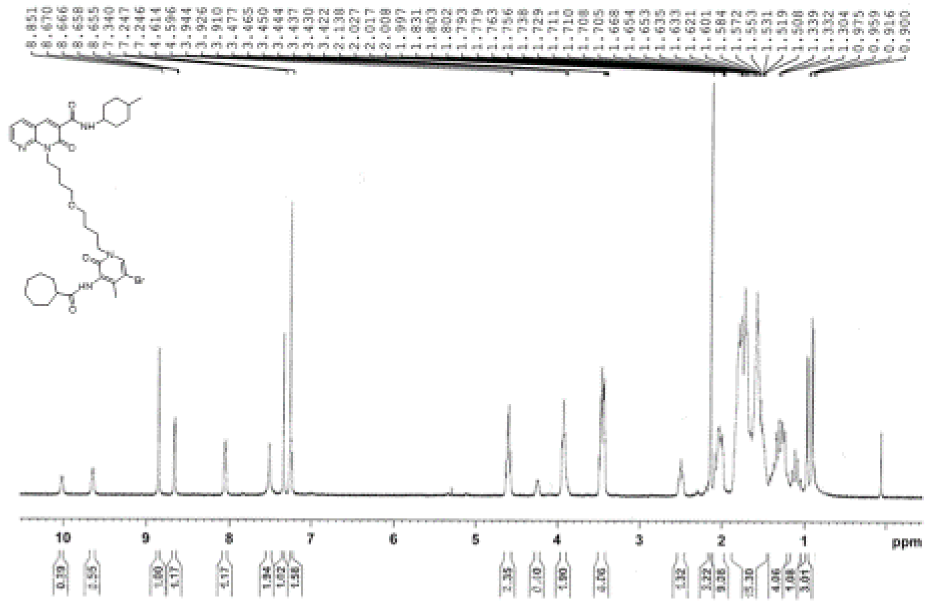

JR60a ^{13}C-NMR

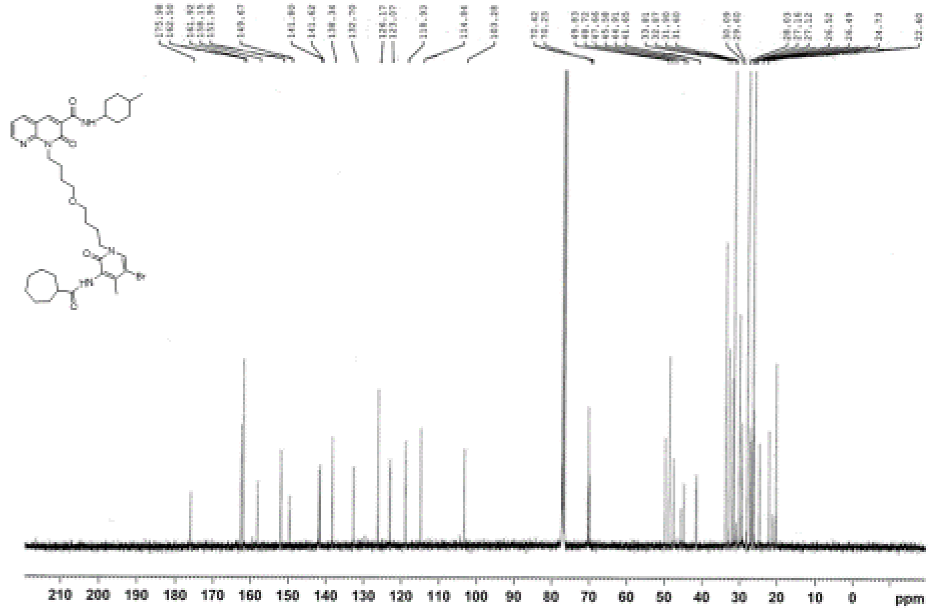

Supplement: Supplementary file 1 [file DataSheet1.pdf]
